# Negative Thermal Expansion in the Materials With Giant Magnetocaloric Effect

**DOI:** 10.3389/fchem.2018.00438

**Published:** 2018-09-25

**Authors:** Fengxia Hu, Feiran Shen, Jiazheng Hao, Yao Liu, Jing Wang, Jirong Sun, Baogen Shen

**Affiliations:** ^1^Beijing National Laboratory for Condensed Matter Physics and State Key Laboratory of Magnetism, Institute of Physics, Chinese Academy of Sciences, Beijing, China; ^2^School of Physical Sciences, University of Chinese Academy of Sciences, Beijing, China

**Keywords:** negative thermal expansion, thermal compensation, giant magnetocaloric effect, spin-lattice coupling, magnetostructural transition

## Abstract

Negative thermal expansion (NTE) behaviors in the materials with giant magnetocaloric effects (MCE) have been reviewed. Attentions are mainly focused on the hexagonal Ni_2_In-type MM'X compounds. Other MCE materials, such as La(Fe,Si)_13_, RCo_2_, and antiperovskite compounds are also simply introduced. The novel MCE and phase-transition-type NTE materials have similar physics origin though the applications are distinct. Spin-lattice coupling plays a key role for the both effect of NTE and giant MCE. Most of the giant MCE materials show abnormal lattice expansion owing to magnetic interactions, which provides a natural platform for exploring NTE materials. We anticipate that the present review can help finding more ways to regulate phase transition and dig novel NTE materials.

## Introduction

Magnetic cooling technique based on magnetocaloric effect (MCE) has attracted an increasing attention for its environmental-friendly and energy-saving superiority compared to conventional vapor compression technique (Annaorazov et al., 1996; Pecharsky and Gschneidner, 1997; Hu et al., 2000a, 2001; Wada and Tanabe, 2001; Tegus et al., 2002; Fujita et al., 2003; Gschneidner et al., 2005; Krenke et al., 2005; Shen et al., 2009; Liu E. K. et al., 2012; Liu J. et al., 2012; Wu et al., 2015). The discovery of giant MCE materials, such as FeRh (Annaorazov et al., 1996), Gd_5_Si_2_Ge_2_ (Pecharsky and Gschneidner, 1997; Gschneidner et al., 2005), La(Fe,Si)_13_ (Hu et al., 2001; Fujita et al., 2003; Shen et al., 2009), MnFeP(As,Ge) (Tegus et al., 2002), MnAs (Wada and Tanabe, 2001), NiMn-based Heusler alloys (Hu et al., 2000a; Krenke et al., 2005; Liu J. et al., 2012), Ni_2_In-type MM'X compounds (Liu E. K. et al., 2012; Wu et al., 2015), has promoted the developing of solid-state magnetic refrigeration technique. A common feature of these materials is the strong spin-lattice coupling. In other words, magnetic and structural transition concurrently takes place, and the change of lattice during the transition does contribute to the giant MCE. Note that a number of MCE materials show abnormal lattice expansion owing to magnetic interactions, which provides a natural platform for exploring negative thermal expansion materials. However, not all the giant MCE materials show negative thermal expansion (NTE), some of them still show positive thermal expansion (PTE), depending entirely on the characteristics of the magnetostructural/magnetoelastic transition and the specific physical origin in different systems.

For example, FeRh alloy shows giant MCE. It crystallizes in the CsCl-type cubic structure and experiences PTE with volume change of Δ*V/V*~ + 0.9% along with the AFM-FM (antiferromagnetic-ferromagnetic) transition without the change of space group. Gd_5_Si_2_Ge_2_ also exhibits giant MCE (Pecharsky and Gschneidner, 1997), which originates from a coupled magnetostructural transition in which slabs of a well defined arrangement of R and T atoms shift ~0.5Å with respect to one another along the *a*-axis when the transition occurs under the influence of temperature (Gschneidner et al., 1997; Morellon et al., 2000). Such a shift gives rise to a PTE with volume change of Δ*V/V* ~ + (0.4–1)% depending on the compositions. MnFeP_1−x_As_x_ (0.15 < x < 0.66) alloys with giant MCE crystallize in hexagonal Fe_2_P-type structure and undergo a first-order FM-PM (paramganetic) magnetic transition around room temperature (Tegus et al., 2002). The space group (*P*$\overset{-}{6}$ 2*m*) keeps unchanged upon the magnetic transition, but the lattice shows an anomaly expansion in the direction of the *a* and *b* axes and a contraction of the *c* axis as the FM phase is formed, which originates from an increase in the intralayer metal-metal bond distance (Liu et al., 2009). As a result, a PTE with Δ*V/V* ~ + 0.06% was observed with increasing temperature. The FM Heusler alloy Ni_2_MnGa with L2_1_ structure shows a giant MCE owing to the martensitic magnetostructural transformation from FM austenitic to FM martensitic phase (Wada and Tanabe, 2001). On heating, the alloy displays a PTE with Δ*V/V* ~ + 1%.

However, the La(Fe_1−x_Si_x_)_13_-based materials with giant MCE show NTE with Δ*V/ V*~ – (1.0–1.6)% around T_C_, depending on compositions, due to the itinerant ferromagnetic nature of the materials (Shen et al., 2009). The space group (Fm$\overset{-}{3}$ c) keeps unchanged across the magnetic transition. Moreover, the hexagonal MnCoGe/MnNiGe-based compounds also show giant MCE and NTE. The optimal compositions undergo a magnetostructural transition from a Ni_2_In-type hexagonal PM phase (space group *P6*_3_*/mmc*) to a TiNiSi-type orthorhombic FM phase (space group *Pnma*). The c axis of hexagonal phase expands while the a-axis contracts upon the martensitic structural transition. As a result, NTE as large as Δ*V/V* = –(2.68–3.9)% occurs depending on compositions (Liu E. K. et al., 2012; Wu et al., 2015; Zhao et al., 2015).

Materials with precise thermal expansion or zero thermal expansion (ZTE) are in urgent demand in modern industry (Rathmann et al., 1968; Namba et al., 2001; Du et al., 2016), such as engineered components, optical mirrors, and printed circuit boards. Generally, ZTE can be reached by combining the materials with PTE and NTE coefficients. To meet various demands, lots of efforts have been made to search for NTE materials, and a number of materials have been discovered showing giant NTE, e.g., ZrW_2_O_8_ (Mary et al., 1996), CuO nanoparticles (Zheng et al., 2008), (Bi,La)NiO_3_ (Azuma et al., 2011), PbTiO_3_-based compounds (Chen et al., 2011), antiperovskite manganese nitrides (Takenaka and Takagi, 2005; Iikubo et al., 2008; Sun et al., 2010a; Song et al., 2011; Wang et al., 2012; Lin et al., 2015), La(Fe,Co,Si)_13_ (Huang et al., 2013), MnCoGe-based materials (Zhao et al., 2015), and reduced Ca_2_RuO_4_ (Takenaka et al., 2017). Among these NTE materials, the phase-transition-type materials (Takenaka and Takagi, 2005; Iikubo et al., 2008; Sun et al., 2010a; Song et al., 2011; Huang et al., 2013; Zhao et al., 2015) have attracted specific attention.

In this paper, we mainly review the negative thermal expansion behaviors based on magnetostructural or magnetoelastic transition in the materials showing giant magnetocaloric effect. Here, the magnetostructural transition refers to the concurrent magnetic and structural transition where the lattice structure (space group) changes during the transition, while the magnetoelastic transition refers to the concurrent magnetic and elastic transition where the lattice structure (space group) remains unchanged but lattice parameter changes elastically.

## La(Fe,Si)_13_-based compounds

### Crystal structure

**Figure 1:**
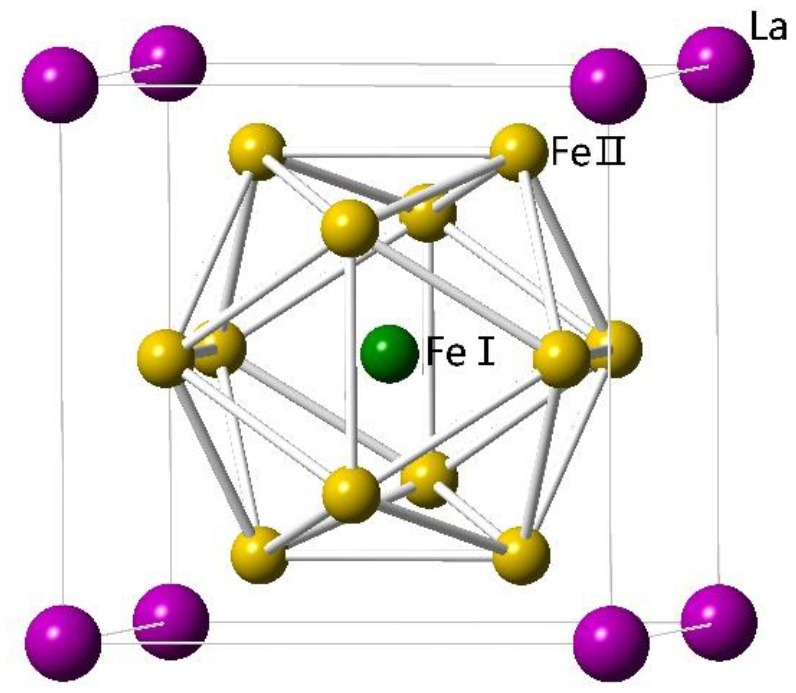
Crystal structure of NaZn_13_-type La(Fe,X)_13_ compounds.

LaFe_13_ does not exist due to the positive formation enthalpy between La and Fe. To get LaFe_13_-based alloys, a third element is needed. The first stable LaFe_13−x_M_x_ compounds were made by Krypiakewytsch et al. (1968) in 1968 when Fe was partially replaced by Si or Al. The compounds show cubic NaZn_13_-type structure with space-group of Fm$\bar{3}$c. In the structure, La atoms occupy the 8a site, while Fe atoms occupy the 8b and 96i sites. Fe atoms at these two sites are denoted as Fe^I^ and Fe^II^, respectively. Al or Si randomly substitute for Fe^II^ site. Fe atoms at Fe^I^ site and La form a CsCl structure. The Fe^I^ are surrounded by an icosahedron of 12 Fe^II^, and the Fe^II^ are surrounded by nine nearest Fe^II^ and one Fe^I^, as shown in Figure 1.

### Magnetocaloric effect

**Figure 2:**
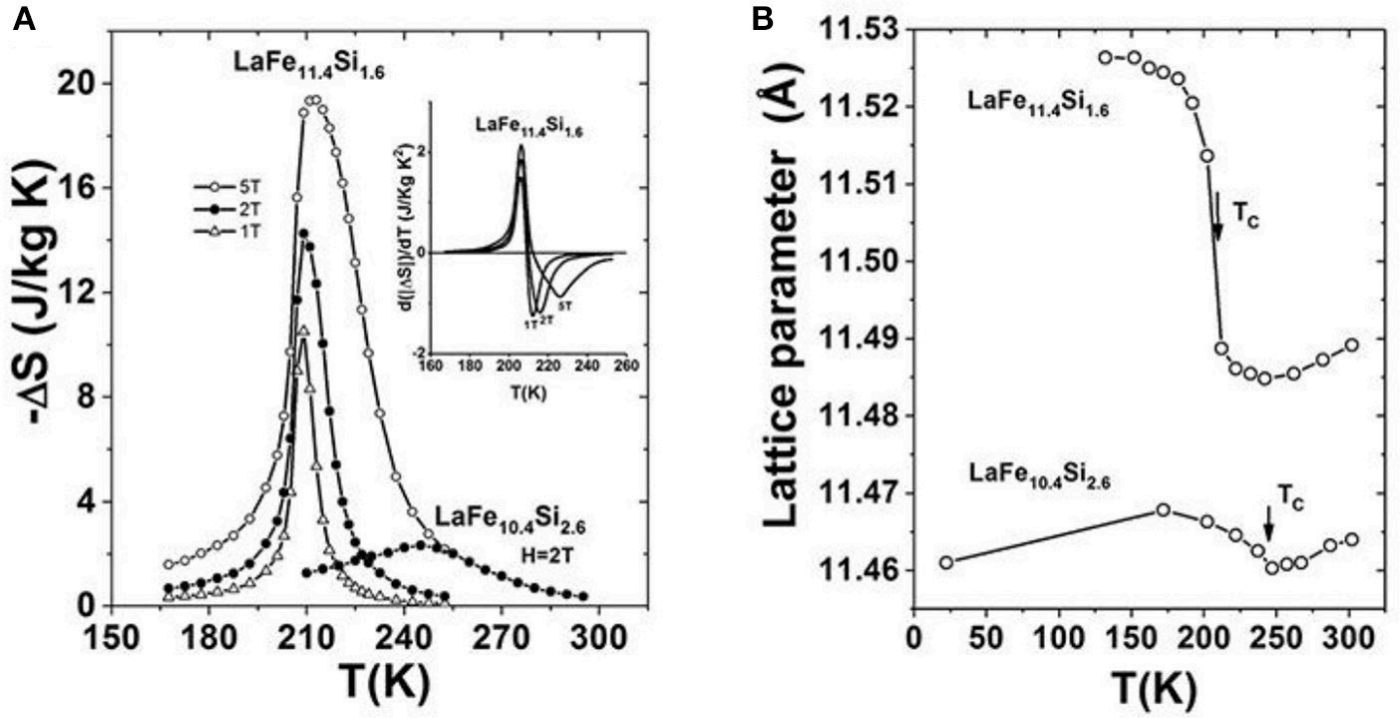
**(A)** Magnetic entropy change –ΔS of LaFe_11.4_Si_1.6_ compared with LaFe_10.4_Si_2.6_. The differential curves of –ΔS for the former is given in the inset. **(B)** Temperature dependence of lattice parameter of LaFe_11.4_Si_1.6_ compared to that of LaFe_10.4_Si_2.6_. Reprinted with permission from Hu et al. (2001).

By analyzing phase formation rule of NaZn_13_-type compounds, LaFe_13−x_Si_x_ compounds with Si content ranging from *x* = 1.2 to 2.4 were successfully synthesized (Hu, 2002; Shen et al., 2009). In 2001, we firstly reported the large MCE in LaFe_11.4_Si_1.6_, originating from a negative lattice expansion and a field-induced itinerant-electron metamagnetic transition (Hu et al., 2001). The maximal magnetic entropy change, –ΔS, reaches 19.4 J kg^−1^K^−1^ around 213 K under a magnetic field change of 0-5T, largely exceeding that of LaFe_10.4_Si_2.6_ with typical second-order transition, as shown in Figure 2A. The differential curves given in the inset of Figure 2A clearly illustrates the asymmetrical broadening of –ΔS peak caused by the field-induced itinerant-electron metamagnetic transition. Figure 2B displays the comparison of lattice change around phase transition between LaFe_11.4_Si_1.6_ and LaFe_10.4_Si_2.6_. One can notice, for LaFe_11.4_Si_1.6_, the negative expansion of lattice parameter can be as much as −0.4%.

**Figure 3:**
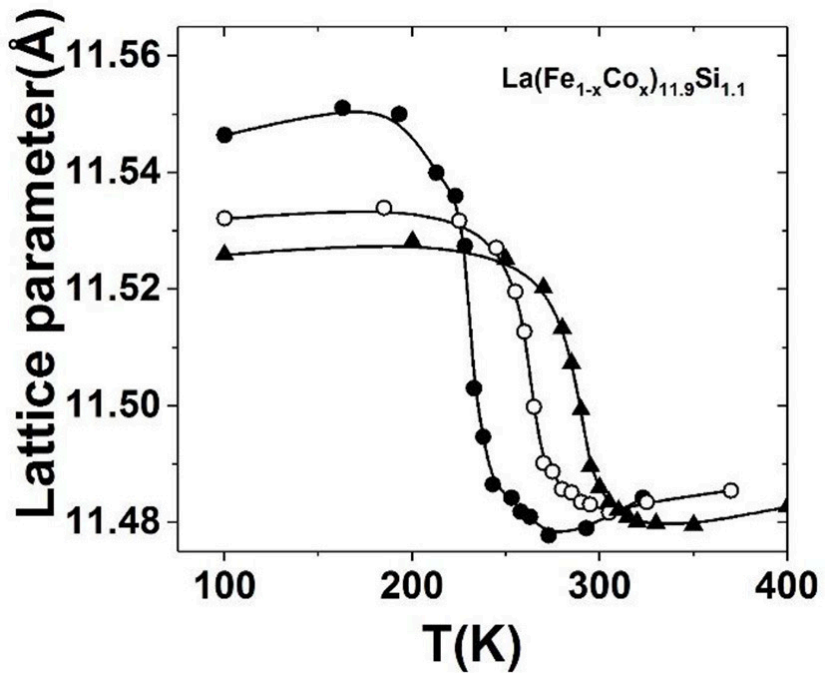
The temperature dependence of lattice parameter for La(Fe_1−x_Co_x_)_11.9_Si_1.1_ (*x* = 0.04, 0.06, 0.08) compounds. Reprinted with permission from Hu et al. (2005).

Introducing the substitution of Co for Fe or interstitial atoms C/H can both impact the exchange coupling between the magnetic elements. This fact can adjust the temperature of phase transition in a wide temperature range covering room temperature while a large MCE is maintained. The maximal room temperature –ΔS can reach 12 J kg^−1^K^−1^ and 20 J kg^−1^K^−1^ under 2 T and 5 T magnetic fields, respectively, for the typical La(Fe,Si)_13_-based compounds (Hu et al., 2002; Shen et al., 2009). The large MCE make the La(Fe,Si)_13_-based compounds attractive to be as room temperature refrigerants. For La(Fe_1−x_Co_x_)_11.9_Si_1.1_ (Hu et al., 2002, 2005), the –ΔS is one times larger than the conventional refrigerant Gd, and is comparable or even exceeds that of Gd_5_Si_2_Ge_2_, the milestone MCE material discovered in 1997 (Pecharsky and Gschneidner, 1997). The large MCE is closely related to negative lattice expansion across phase transition, which can be identified in the temperature dependence of lattice parameter for La(Fe_1−x_Co_x_)_11.9_Si_1.1_ (*x* = 0.04, 0.06, 0.08) compounds (Figure 3).

### Negative thermal expansion behaviors

Although the large MCE of La(Fe,Si)_13_-based compounds owing to the significant negative lattice expansion was discovered in the early 2000 (Hu et al., 2000b; Shen et al., 2009), these materials have never been considered as negative thermal expansion (NTE) materials till 2013. As NTE materials, Huang et al. firstly reported the giant NTE in La(Fe,Si,Co)_13_ compounds (Huang et al., 2013).

For LaFe_13−x_Si_x_, the NTE properties were found to be strongly dependent on the Si contents. A sharp volume change from 170 to 240 K was observed in LaFe_11.5_Si_1.5_ with a NTE temperature window (ΔT) about 70 K. The estimated ΔL/L is 3.5 × 10^−3^, which is comparable to those of Mn_3_ZnN (4.6 × 10^−3^) (Takenaka and Takagi, 2005) and Mn_3_GaN (3.8 × 10^−3^) (Takenaka et al., 2008). With increasing Si content from x = 1.5 to 2.4, the linear expansion becomes broader gradually, leading to a large negative slope in a wider temperature range. This result illustrates that the replacement of Fe by Si can broaden the temperature window of NTE. However, the numerical values of NTE coefficient rapidly drop with the increase of Si content, which is an unfavorable character for practical applications.

**Figure 4:**
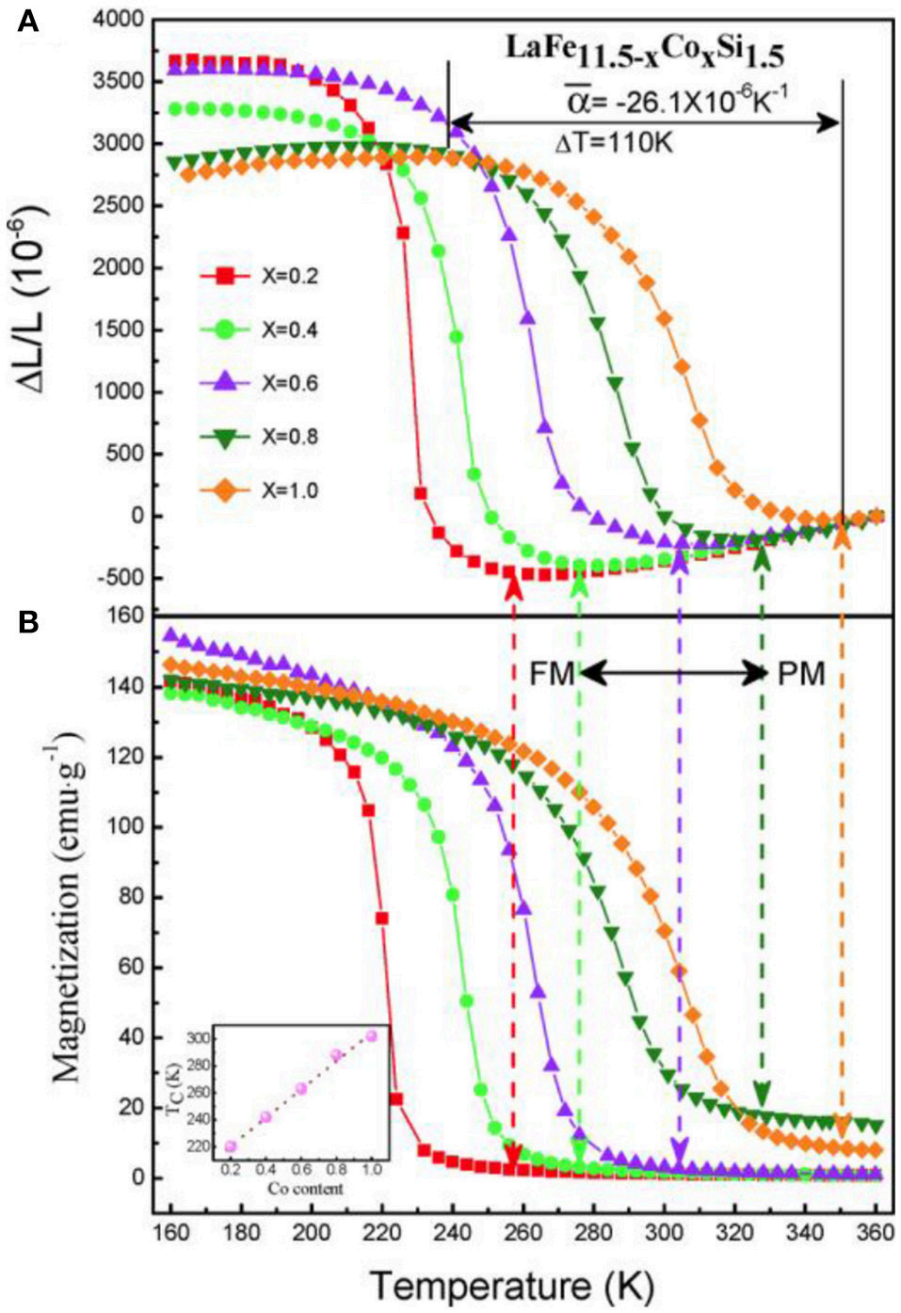
Temperature dependence of **(A)** linear thermal expansions ΔL/L (reference temperature: 360 K) compared to **(B)** the magnetization for LaFe_11.5−x_Co_x_Si_1.5_ (*x* = 0.2, 0.4, 0.6, 0.8, and 1.0). Reprinted with permission from Huang et al. (2013).

To improve the performance of NTE behavior, the substitution of Co for Fe was introduced. Figure 4 displays linear thermal expansion as a function of temperature and its comparison with magnetization for LaFe_11.5−x_Co_x_Si_1.5_. Increasing Co content broadens the temperature window, ΔT, of NTE, and drives it to a higher temperature. The ΔT width of NTE is 74 K, 90 K, and 110 K for *x* = 0.2, 0.4, and 1.0, respectively. More importantly, the change of ΔL/L within the NTE window reduces less rapidly with Co compared to the case with Si. The composition with *x* = 0.10 produces an average coefficient of thermal expansion (CTE) of $\overset{-}{\alpha }$ = −26.1 × 10^−6^ K^−1^ from 240 K to 350 K, covering room temperature. This numerical value of $\overset{-}{\alpha }$ is about 3 times larger than that of the commercial NTE materials ZrW_2_O_8_ with α = −9 × 10^−6^ K^−1^. Moreover, the absolute value of the negative CTE reaches those of high-expansion metals such as Al (α = 23 × 10^−6^ K^−1^ at room temperature). These findings suggest the potential application of La(Fe,Si)_13_-based compounds as NTE materials.

More interestingly, zero thermal expansion (ZTE) has been recently reported in La(Fe,Si)_13_-based compounds in a wide temperature range away from phase transition (Li et al., 2017), which is a fascinating phenomenon in these compounds.

## Ni_2_In-type MM'X compounds

### Crystal structure

Hexagonal Ni_2_In-type MM'X (M, M' = transitional element, *X* = main element) compounds have attracted lots of attention owing to the rich magnetic and structural behaviors. Ferromagnetic or antiferromagnetic coupling occurs depending on the compositions and atomic local environments (Niziol et al., 1981; Caron et al., 2011; Gercsi et al., 2011; Liu E. K. et al., 2012). As a member of family, the stoichiometric MnCoGe alloy undergoes diffusionless martensitic structural transition, *T*_stru_, from Ni_2_In-type hexagonal (space group: P6_3_/mmc) to TiNiSi-type orthorthombic (space group: Pnma) structure at *T*_stru_ ~420 K and a transition of ferromagnetic ordering at *T*_*c*_~345 K (Niziol et al., 1981). Both the martensitic and austenitic phases display ferromagnetic nature, and the orthorhombic phase shows a higher magnetic moment *M*_s_ ~4.13 μ_B_ than the hexagonal phase (*M*_s_ ~2.76 μ_B_) (Niziol et al., 1981). On the other hand, the stoichiometric MnNiGe undergoes a similar martensitic structural transformation at *T*_*stru*_~470 K, and a magnetic transition at *T*${}_{N}^{M}$ ~346 K. The martensitic and austenitic phases have distinct magnetic structure. The former shows spiral antiferromagnetic (AFM) coupling with Neel temperature *T*${}_{N}^{M}$ at 346 K while the latter exhibits ferromagnetic (FM) nature with intrinsic Curie temperature *T*${}_{C}^{A}$ at 205 K (Niziol et al., 1981).

**Figure 5:**
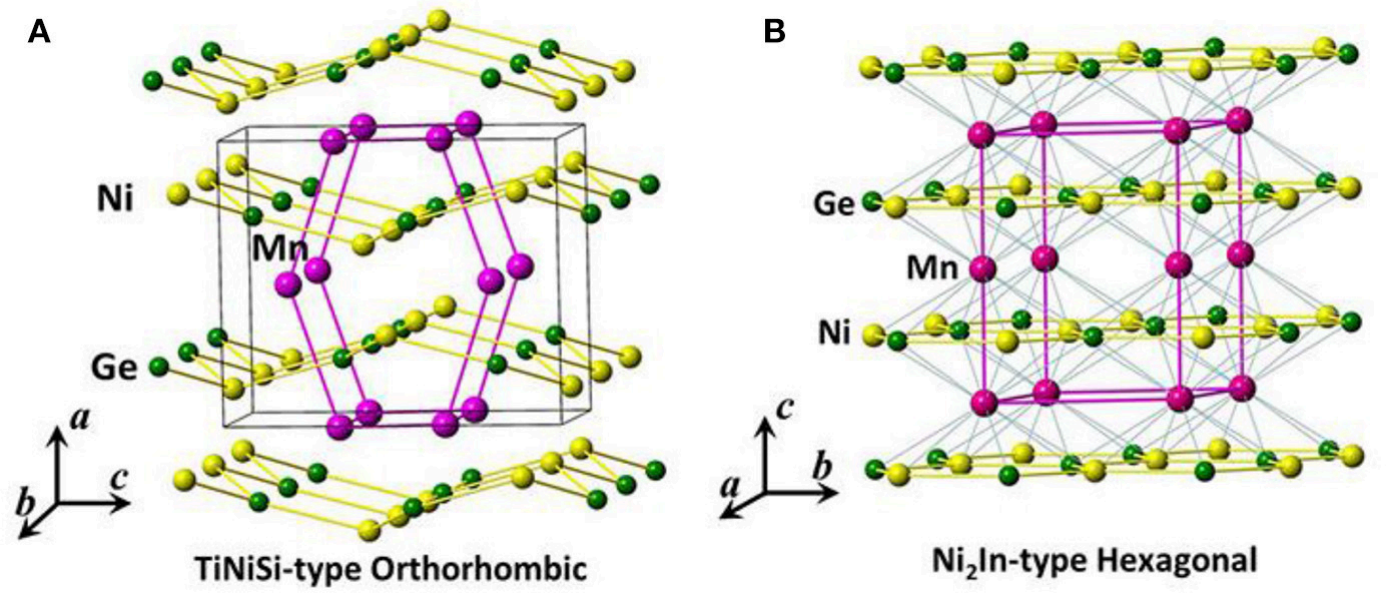
The sketches of **(A)** orthorhombic martensitic and **(B)** hexagonal austenitic structures. The black lines in **(A)** enclose one unit cell of TiNiSi-type orthorhombic structure while the purple lines in **(B)** enclose the one of Ni_2_In-type hexagonal structure. The unit cell of hexagonal phase transforms into the zigzag-type structure in the orthorhombic phase, as indicated by purple lines in **(A)**.

Figure 5 shows the sketches of orthorhombic martensitic and hexagonal austenitic structures for MnCoGe (Wu et al., 2015).

### Magnetocaloric and barocaloric effects

For the MM'X compounds, austenitic phase has a smaller volume than martensitic phase. This fact prompts one to think that the introduction of smaller atoms or vacancies may stabilize the austenitic phase and shift *T*_*stru*_ to a lower temperature. Following this way, magnetostructural transition has been indeed realized by introducing smaller atoms or vacancies, such as MnCo_1−x_Ge (Wang et al., 2006), Mn_1−x_CoGe (Liu et al., 2010), Mn_1−x_Cr_x_CoGe (Caron et al., 2011). Moreover, It has demonstrated that valence electron concentration (*e/a*) can also affect *T*_*stru*_. By introducing Indium (In) atom (2.00 Å, 5s^2^5p^1^) with a larger radius but fewer *e/a* to replace Mn (1.79 Å, 3d^5^4s^2^), Co (1.67 Å, 3d^7^4s^2^), or Ge (1.52 Å, 4s^2^4p^2^), magnetostructural transition is also created in (Liu E. K. et al., 2012). As a result, large MCE has been observed in these MnCoGe-based materials.

On the other hand, it has been found that, for MnNiGe, the AFM coupling in the martensitic structure is not robust. The replacements of Mn or Ni by Fe atom can convert the AFM into FM coupling and stabilize the austenitic phase (Liu E. K. et al., 2012). As a result, the *T*_*stru*_ shifts to low temperature, and magnetostructural transition is realized and hence large MCE appears in an extended temperature window from 350 K down to 70 K in MnFeNiGe alloys.

**Figure 6:**
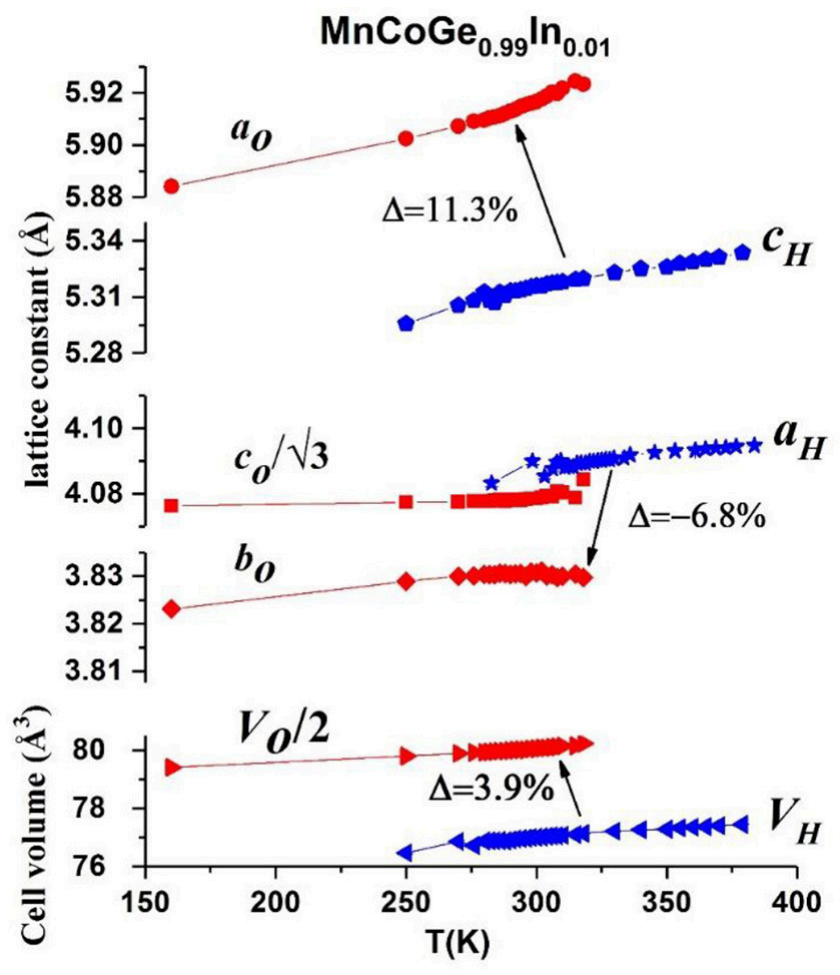
Lattice constants and unit-cell volume of hex. and orth. structure as a function of temperature for MnCoGe_0.99_In_0.01_. The blue and red symbols correspond to the hex. and orth. structure, respectively. The red circle, red square and red rhombus stand for a_o_, c_o_/√3 and b_o_, respectively. The blue pentagon and blue star represent the c_h_ and a_h_, respectively. The unit-cell volume of hex. and half of unit-cell volume of orth. structure are symbolized as blue and red triangle, respectively. Reproduced under a Creative Commons Attribution 4.0 International License from Wu et al. (2015).

Moreover, giant barocaloric effect has been demonstrated in MnCoGe_0.99_In_0.01_ compound (Wu et al., 2015). Neutron powder diffraction (NPD) studies indicated that the lattice of hexagonal phase expands significantly along the c-axis (*c*_*H*_) by 11.3% while contracts along the a-axis (*a*_*H*_) by −6.8% during the magnetostructural transformation, *T*_*mstru*_. As a result, negative lattice expansion as large as Δ*V/V* = *(V*_ortho_/2-*V*_hex_)/*V*_hex_~ −3.9% occurs for MnCoGe_0.99_In_0.01_, as shown in Figure 6. Note that the unit cell volume and lattice constants of hexagonal and orthorhombic phases have relations as *a*_*o*_ = *c*_*H*_, *b*_*o*_ = *a*_*H*_, *c*_*o*_ = √*3a*_*H*_ and *V*_*O*_ = 2*V*_*H*_.

The calorimetric measurements using a differential scanning calorimeter (DSC) revealed that the total change of transition entropy can be as large as 55 J kg^−1^K^−1^ contributed by the large difference of internal energy across the transition for MnCoGe_0.99_In_0.01_. High resolution NPD studies under hydrostatic pressure revealed that pressure can shift the *T*_*mstr*_ to lower temperature at a rate of 7.7 K/kbar. The large latent heat and high sensitivity of the magnetostructural transition to pressure result in giant inverse barocaloric effect (Wu et al., 2015). The entropy change, ΔS, and adiabatic temperature change, ΔT_ad_, under a pressure of 3 kbar reaches 52 J kg^−1^K^−1^ and −18 K, which exceed those of most materials, including the giant magnetocaloric effect driven by a magnetic field of 5 T (Gschneidner et al., 2005) that can be only available by superconducting magnets. The refrigeration cooling power (RCP) reaches 1190 J kg^−1^.

### Negative thermal expansion behavior

Similar to the case of La(Fe,Si)_13_-based compounds, the Ni_2_In-type MM'X compounds have rarely been considered as negative thermal expansion (NTE) materials because of the limited temperature region of phase transition, though they surely have significant negative lattice expansion and large MCE and barocaloric effect owing to the magnetostructural transition.

#### Giant negative thermal expansion in MnCoGe-based materials

In 2015, Zhao et al. firstly reported giant NTE in bonded MnCoGe-based materials (Zhao et al., 2015). The as-prepared compounds are very brittle and even collapse into powders during preparation. Through introducing a few amounts (3–4 wt%) of epoxy to bond the powders, residual stress was introduced, hence the structural transition was broadened due to the lattice softening enforced by the stress. Hence, giant NTE has been achieved in a wide temperature region. Moreover, excellent mechanical properties and tunable electrical conductivity has been demonstrated for the bonded powders.

Neutron powder diffraction (NPD) studies indicate that the change of lattice volume during the magnetic transition can be as much as 3.9, 3.9, and 3.8% for MnCoGe_1−x_In_x_ (*x* = 0.01, 0.02), and MnCoGe_0.97_Sb_0.03_, respectively. Accordingly, the evaluated linear expansion is equal to ΔL/L ~10,995 × 10^−6^ and the average NTE coefficient is about $\overset{-}{\alpha }$ ~ −183.3 × 10^−6^/K if the polycrystalline powder is supposed to expand isotropically for MnCoGe_0.99_In_0.01_.

**Figure 7:**
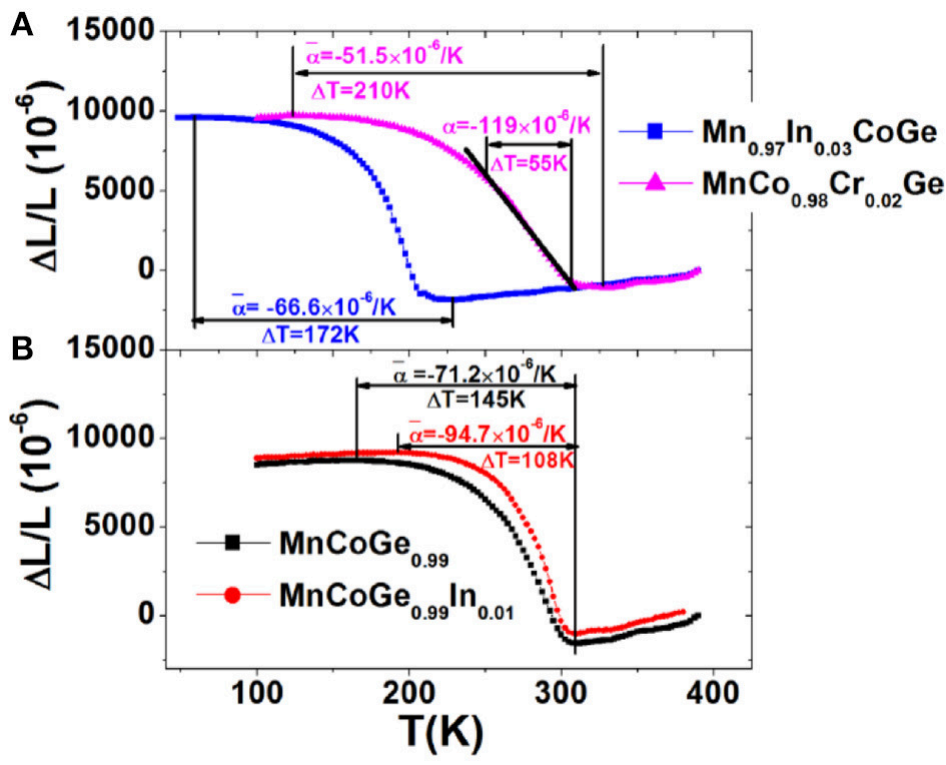
Temperature dependent linear thermal expansions ΔL/L (the reference temperature is 390 K) for bonded samples with various compositions **(A)** Mn_0.97_In_0.03_CoGe, MnCo_0.98_Cr_0.02_Ge, and **(B)** MnCoGe_0.99_, MnCoGe_0.99_In_0.01_. Reprinted with permission from Zhao et al. (2015).

Linear expansion, ΔL/L, was measured using high resolution strain gauge for the bonded plates. It was found that the ΔL/L behaves isotropically, independent of the in-plane measured axis. Figure 7 shows the ΔL/L as a function of temperature for some typical compositions. The maximal ΔL/L keeps above 10,231 × 10^−6^, while the average linear NTE coefficient ranges from −51.5 × 10^−6^/K to −94.7 × 10^−6^/K in the tunable temperature region from ~60 to 330 K covering room temperature for the bonded MnCoGe-based materials with different compositions. Because of the significant broadening of phase transition caused by residual stress and the possibly porosities introduced, the ΔL/L and $\overset{-}{\alpha }$ are somewhat smaller than those estimated from the X-ray diffraction (XRD) or NPD measurements. However, the maximal ΔL/L measured has reached 93% of the crystallographic value. These novel NTE properties are over the performance of most other reported materials previously. For example, the average $\overset{-}{\alpha }$ ~ −51.5 × 10^−6^/K with operation temperature window as wide as 210 K was observed in MnCo_0.98_Cr_0.02_Ge, which is more than 5 times larger than that of the commercial NTE materials ZrW_2_O_8_ with $\bar{\alpha }$ = −9 × 10^−6^/K.^1^ More interestingly, in the temperature region from 250 to 305 K covering room temperature, the CET α (−119 × 10^−6^/K) remains nearly independent of temperature (see Figure 7A), and this value is about 40% larger than the that (−82 × 10^−6^ /K) of Bi_0.95_La_0.05_NiO_3_ (320–380 K) (Azuma et al., 2011), and more than four times larger than the α (−25 × 10^−6^ /K) of an antiperovskite manganese nitride (316–386 K) (Takenaka and Takagi, 2005).

**Figure 8:**
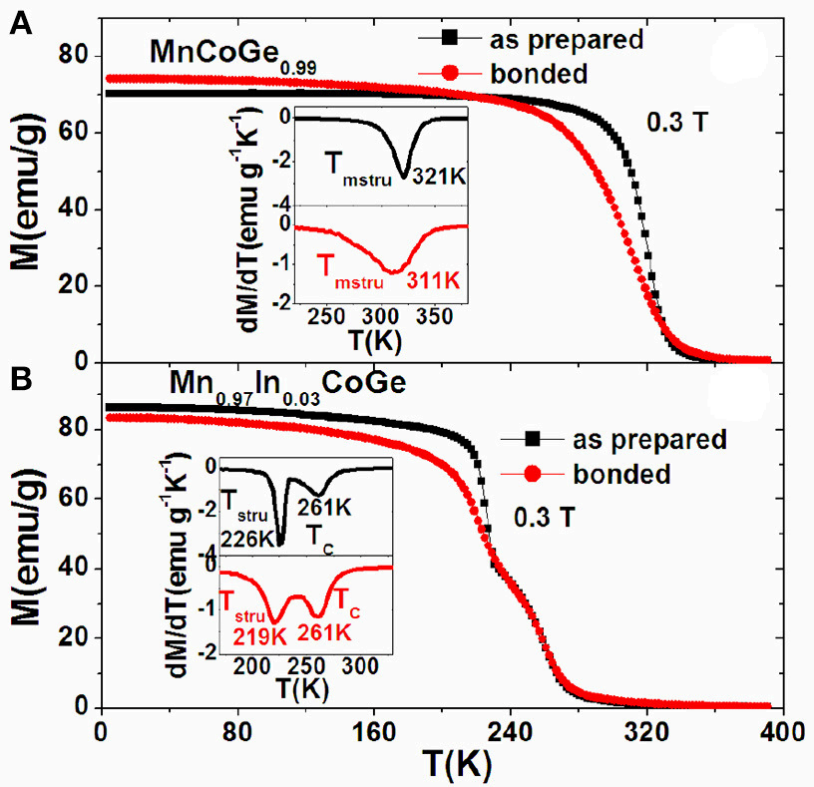
The comparison of temperature dependent magnetization under 0.3T for the bonded and as-prepared compositions **(A)** MnCoGe_0.99_ with magnetostructural coupling and **(B)** Mn_0.97_In_0.03_CoGe with decoupled *T*_*stru*_ and *T*_*C*_. The insets show corresponding dM/dT plots. Reprinted with permission from Zhao et al. (2015).

Figures 8A,B presents the temperature dependence of magnetization (M-T curves) measured under a 0.3 T magnetic field for the bonded and as-prepared compositions with and without magnetostructural coupling. From Figure 8 and the dM/dT plots in the insets, one can distinguish that the structural/magnetostructural transformation (*T*_*stru*_/*T*_*mstru*_) was significantly broadened while the pure magnetic transition keeps unchanged around *T*_*C*_ for the bonded samples. For the bonded MnCoGe_0.99_ with magnetostructural transition, the *T*_*mstru*_ shifts by 10 K to low temperature, while for the Mn_0.97_In_0.03_CoGe with decoupled *T*_*stru*_ and *T*_*C*_, the *T*_*stru*_ shifts by 7 K to low temperature while *T*_*C*_ keeps unchanged (261 K). The significant broadening of the structural/magnetostructural transformation enforced by the residual stress governs the NTE behavior in the bonded samples.

From viewpoint of industry, electrical conductivity is a critical parameter for practical applications. For the bonded MnCoGe-based materials, the electrical conductivity can be adjusted through choosing distinct binders. It has been experimentally demonstrated that the electrical resistivity for the materials with Ag-epoxy as binder is lower than the one with epoxy as binder by 3 orders of magnitude. Moreover, the mechanical properties have been largely improved. All these suggest the high potential of MnCoGe-based compounds as NTE materials, which can be used to compensate the materials with high PTE, such as the popularly-used organic or plastic materials.

#### Ultra-low thermal expansion realized in MnCoGe_0.99_In_0.01_ through self-compensation

The martensitic structural transition in Ni_2_In-type MM'X compounds behaves extremely sensitive to pressure compared to magnetic field (Wu et al., 2015). The introduced residual strain and defects during pulverization (Wu et al., 2016) or cold pressing (Liu et al., 2015) can also affect the magnetostructural coupling besides hydrostatic pressure. It has been reported that the introduced residual strain in the MnCoGe_1−x_In_x_ thin slices prepared by cold pressing can stabilize the austenite phase, as a result, the temperature window of martensitic transformation is broadened, the magnetic and structural transition becomes decoupling (Liu et al., 2015). Similarly, with reducing particle size, austenitic phase becomes stable and a high fraction of austenitic phase loses the martensitic transformation, and retains the hexagonal FM structure in the entire temperature range. Considering the large difference of volume between austenitic and martensitic phases, one may prompt to think that adjusting phase fraction and magnetostructural transition through residual strain can lead to controllable NTE or even ZTE.

Indeed, ultra-low thermal expansion has been realized in the giant NTE material MnCoGe_0.99_In_0.01_ by controlling the crystallinity degree and phase transition (Shen et al., 2017). A high fraction of sample can be converted into amorphous structure by ball milling, which turns to show PTE while the remained crystallites exhibit decreased NTE in an extended temperature window. Ultra-low thermal expansion can be reached and the NTE can be totally adjusted through self-compensation effect.

Samples with different particle sizes were made through energetic ball milling. For small particles, high-resolution TEM (transmission electron microscopy) images indicate the mixture of a high fraction of amorphous structures and nanocrystallites full of atomic defects. The amounts of the amorphous structure were estimated by high-resolution neutron powder diffraction (NPD).

**Figure 9:**
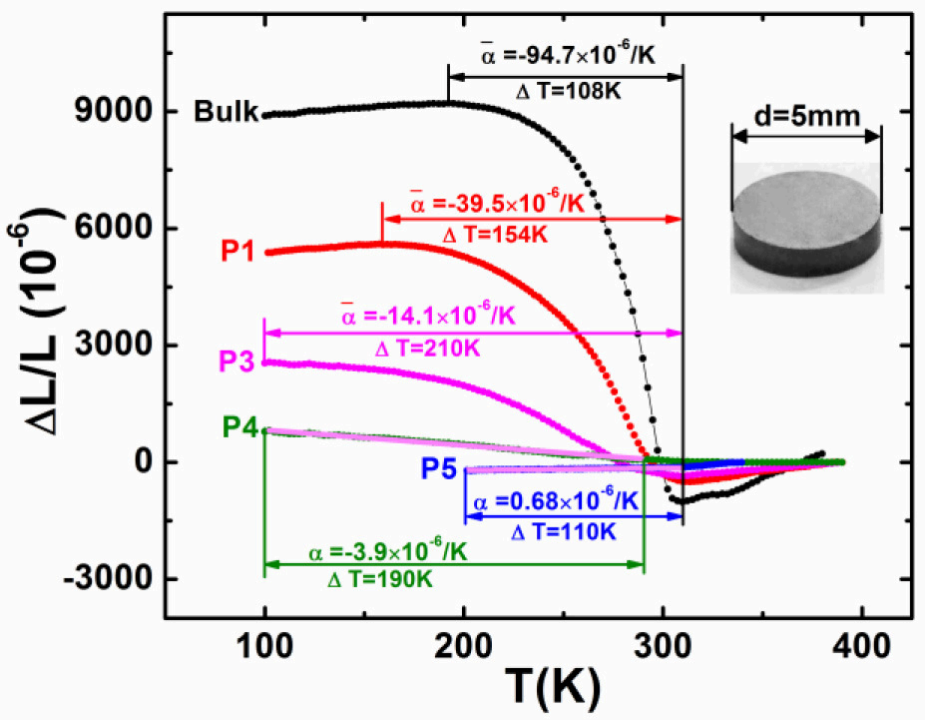
Linear thermal expansion ΔL/L as a function of temperature for the bonded MnCoGe_0.99_In_0.01_ samples with different particle size in comparison to the bulk (the reference temperature is 390 K). Particle size: P1 (10–20 μm), P3 (2–5 μm), P4 (1–2 μm), and P5 (0.3–1 μm). The morphology of the bonded sample is shown in the inset. Adapted from Shen et al. (2017). APL Materials, Vol. 5, Article ID 106102, (2017); licensed under a Creative Commons Attribution (CC BY) license.

Figure 9 shows ΔL/L vs. temperature for the bonded particles compared to the bulk, which was measured by using high-resolution strain gauge. One can notice that the maximal ΔL/L declines while the operating ΔT significantly broadens with reducing particle size. For small particles P3 (2–5 μm) and P4 (1–2 μm), NTE dominates in the entirely measured temperature range from 310 K to 100 K.

For the smallest particles with size P5 (0.3–1 μm), the NTE disappears and turns out to be ultra-low PTE, and the CTE α is as small as + 0.68 × 10^−6^/K from 200 to 310 K. This result suggests that the material can be also useful as a ZTE material, in addition to be used as compensators for PTE material. Moreover, one can notice that the low NTE for P4 (1–2 μm) and the ultra-low PTE for P5 (0.3–1 μm) is nearly independent of temperature in the corresponding temperature region (see the pink lines in Figure 9), which is appreciable for practical applications.

The tunable CTE with particle size originates from the coexistence of crystalline phases and amorphous structure, particularly for the small particles. Variable temperature XRD and neutron NPD studies revealed that the phase ratio changes remarkably with crystallite size. About 7, 14.5, 38.5, 44.8, and 55.2% austenitic phase lost the martensitic transformation and remains the hexagonal structure down to low temperature for the particles P1 (10–20 μm), P2 (5–10 μm), P3 (2–5 μm), P4 (1–2 μm), and P5 (0.3–1 μm), respectively.

**Figure 10:**
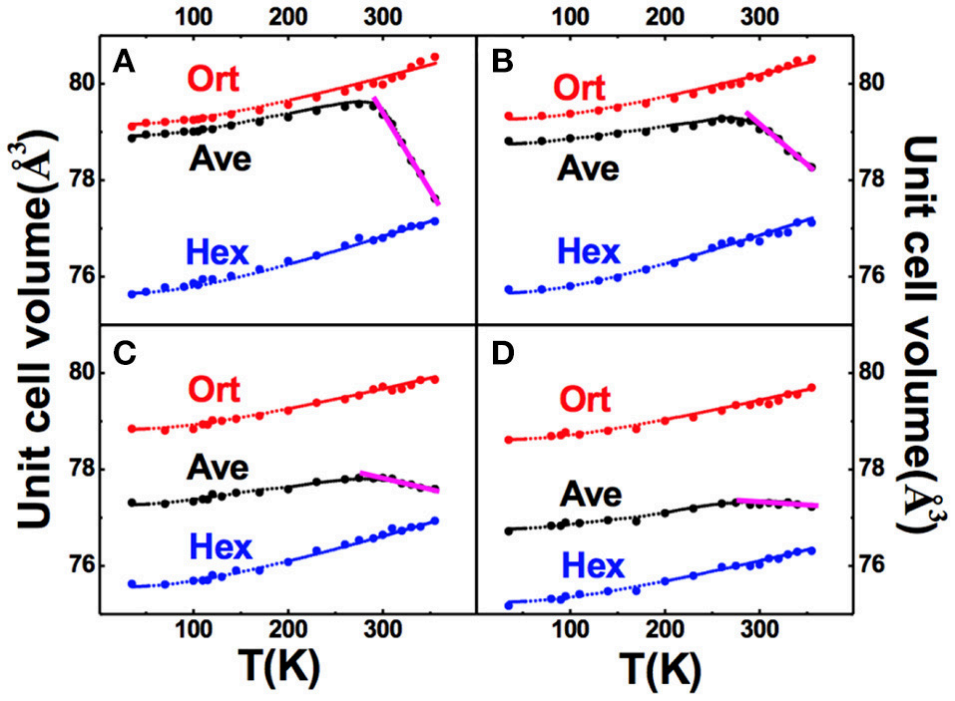
Refined unit cell volumes of martensitic structure (red dot), austenitic structure (blue dot), and the weighted average (black dot), as a function of temperature for the overall crystalline phases of various particles of MnCoGe_0.99_In_0.01_: **(A)** P_1_(10~20 μm), **(B)** P_2_(5~10 μm), **(C)** P_4_(1~2 μm), **(D)** P_5_(0.3~1 μm). The fitting curves according to Grüneisen law are represented by the small dots. Adapted from Shen et al. (2017). APL Materials, Vol. 5, Article ID 106102, (2017); licensed under a Creative Commons Attribution (CC BY) license.

Figure 10 shows the unit cell volume as a function of temperature (*V-T* curve) for the orthorhombic structure (red dot), hexagonal structure (blue dot), and the weighted average volume $\overset{-}{V}$ (black dot) calculated based on the refined phase fraction. Moreover, the temperature dependence of lattice volume was fit based on the Grüneisen law, and the results agree well with the refined results for either orthorhombic or hexagonal phase.

From Figure 10 (black curve), one can notice that PTE occurs below 290 K and turns to be NTE above this point. TEM studies indicated that crystallite size reduces with particle size, and the average crystallite sizes are about 15 nm and 8 nm for P4 (1–2 μm) and P5 (0.3–1 μm), respectively. With reducing the crystallite size, the both PTE and NTE coefficients decline, which are mainly caused by the instability of magnetostructural transformation due to residual stress and defects.

On the other hand, the contribution from amorphous structure cannot be ignored, noting the amount reaches 40% for the smallest particles P5(0.3~1 μm). Detailed analysis indicated that the amorphous structure shows positive thermal expansion (PTE), and its compensation effect plays an essential role in the realization of ultra-low expansion even if the possible contribution from the small fraction of epoxy was considered. Details were given in the Supplementary Material in Shen et al. (2017).

#### Colossal negative thermal expansion in fine-powdered Mn_0.98_CoGe

In 2016, Lin et al. prepared fine-powdered Mn_0.98_CoGe by two ways of high energy ball milling (BM) and repeated thermal cycling (TC) across the martensitic transformation, and compared the NTE behaviors (Lin et al., 2016). It was found that the both ways can make the temperature window (ΔT) of NTE broadening. For the powders experienced TC for ten times, the ΔT broadens to 90 K (309–399 K), and the CTE is about α_*L*_ ~ −141 × 10^−6^/K.

The SEM result showed that the average particle size for the powder of Mn_0.98_CoGe alloy through 10 times of TC treatments is 8.2 μm, and for the powder through 0.5, 5, and 12 h of BM process are 7.4, 3.9, and 2.2 μm, respectively.

**Figure 11:**
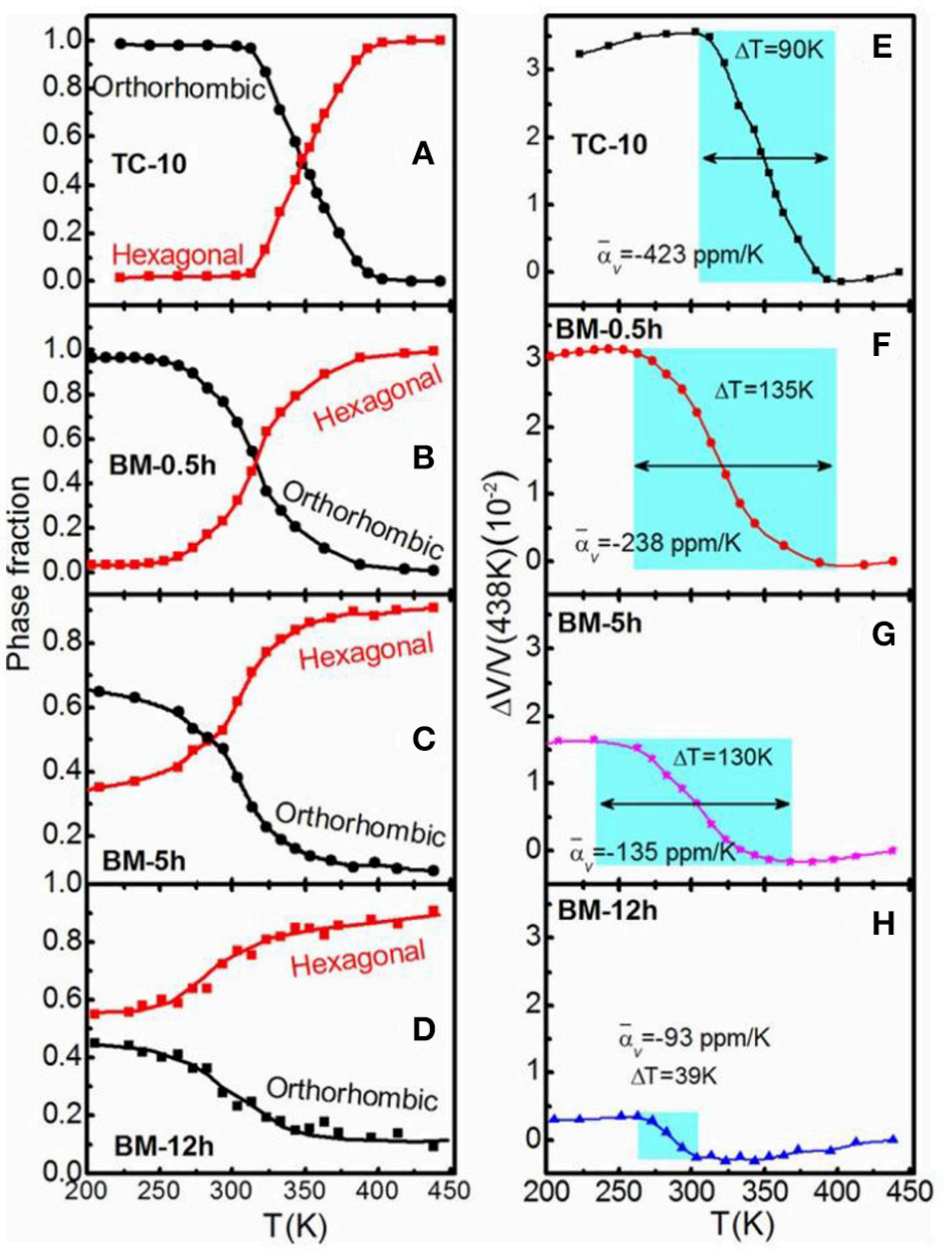
The temperature dependent phase fraction for the Mn_0.98_CoGe samples prepared by **(A)** TC for 10 times, **(B)** BM for 0.5 h, **(C)** BM for 5 h, and **(D)** BM for12 h; The temperature dependent volumetric thermal expansions Δ*V/V* (the reference temperature is 438 K) for the corresponding samples is shown in **(E–H)**, respectively. The NTE temperature region is highlighted for all samples. Reprinted with permission from Lin et al. (2016).

Figures 11A–D display the refined phase fractions for both orthorhombic and hexagonal phases based on the variable temperature XRD patterns for typical samples. For the powders experienced TC for 10 times, the transition of two phases is ~98%. For the powders prepared by BM for 0.5 h, the martensite transformation is broadened, and the transition ratio decreased to ~93%. With increasing BM time to 5 and 12 h, the martensite transformation window was further broadened, and the transition ratio reduced to ~60 and 36%, respectively.

Figures 11E–H show the corresponding volumetric thermal expansion coefficient (α_*V*_) calculated based on the refined phase volume and fraction. For the powders experienced TC for 10 times, NTE effect appears between 309 and 399 K (ΔT = 90 K), and the corresponding α_*V*_ is about −423 × 10^−6^/K. For the powders prepared by BM for 0.5 h, ΔT extends to 135 K (from 258 to 393 K), and α_*V*_ value becomes −238 × 10^−6^/K. Further increasing BM time to 5 and 12 h results in the decreases of both ΔT and α_*V*_, and the α_*V*_ values are about −135 × 10^−6^/K (236–366 K, ΔT = 130 K) and −93 × 10^−6^/K (264–303 K, ΔT = 39 K), respectively. The corresponding linear α_*L*_ values are about −141 × 10^−6^/K (ΔT = 90 K), −79.6 × 10^−6^/K (ΔT = 135 K), −45 × 10^−6^/K (ΔT = 130 K), and −31 × 10^−6^/K (ΔT = 39 K) for the samples experienced TC for 10 times, BM for 0.5 h, BM for 5 h, and BM for 12 h, respectively.

For the samples experienced TC for 10 times, the both temperature window of NTE and the α_*L*_ magnitude are larger than those of typical NTE material Bi_0.95_La_0.05_NiO_3_ (α_*L*_ = −137 × 10^−6^/K, 320–380 K) (Azuma et al., 2011). This α_*L*_ magnitude for the samples experienced TC for 10 times is several times larger than that of common metals (Takenaka et al., 2012) and even larger than that of most polymers (Sullivan and Lukehart, 2005; Chu et al., 2012; Takenaka and Ichigo, 2014).

The NTE behavior has different origins for the samples prepared by the two ways. For the powder prepared by BM, the broadening of temperature window of martensitic transformation originates from the accumulated residual strain. With increasing the BM time, the overall NTE window first expands and then shrinks. For the powder prepared by TC, the sample does not suffer any external pressure, and the introduced strain is thus weaker than the case by BM, hence leading to the completely different evolution of phase transition with temperature.

All these results suggest the TC or slightly BM treatments can both extend temperature interval and optimize the NTE properties, which have high potential applications as the PTE compensators by forming composites.

#### Negative thermal expansion in Mn-Co-Ge-In thin films

In recent years, many materials with magnetostructural transition, such as Gd_5_Si_2_Ge_2_ (Pires et al., 2015), FeRh (Zhou et al., 2013), metamagnetic NiMn-based Heusler alloys (Akkera et al., 2015), and MnAs (Mosca et al., 2008), have been explored in nanostructured thin films, aiming to activate novel properties and explore applications in micro-scale of these materials. Recently, Liu et al. firstly explored the fabrication of MnCoGe-based alloy films, and studied the magnetocaloric effect and thermal expansion behaviors of the films obtained (Liu et al., 2018).

MnCoGe_0.995_In_0.005_ films with thickness of 45 nm were successfully grown on (001)–LaAlO_3_, (001)–SrTiO_3_, and (0001)–Al_2_O_3_ substrates by using pulsed laser deposition (PLD) technique. It was found that the obtained films exhibit three dimensional growth mode, and the island size varies for thin films grown on different substrates. Room temperature XRD patterns imply that the films crystallize in mixed hexagonal and orthorhombic structures, which are textured along the out of plane (00*1*) direction for the hexagonal structure and (*h*00) orientations for the orthorhombic structure. For all the films, there exists obvious thermal hysteresis between the heating and cooling process in a wide temperature range, indicating that first-order magnetostructural transformation was retained. Moreover, the temperature window of the transformation for the films significantly broadened compared to that of the bulk alloy.

**Figure 12:**
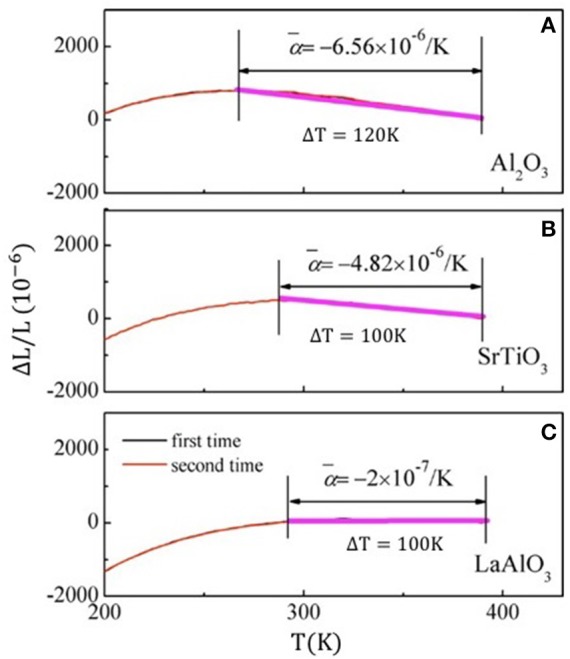
Temperature dependent linear thermal expansion ΔL/L for the Mn-Co-Ge-In thin films grown on Al_2_O_3_ **(A)**, SrTiO_3_ **(B)**, LaAlO_3_ **(C)**. Each has been repeated at least two times. Reprinted with permission from Liu et al. (2018).

The broadened magnetostructural transformation of Mn-Co-Ge-In films can be ascribed to the stress effect and crystalline defects. Besides, the commercially supplied Al_2_O_3_, SrTiO_3_, and LaAlO_3_ substrates have positive linear thermal expansion coefficients + 7.5 × 10^−6^/K, + 9.4 × 10^−6^/K, and + 1 × 10^−5^/K, respectively. Consequently, the different films denote tunable negative thermal expansion due to different island size and strain state of the films along with the compensating effect from substrates. As can be seen from Figure 12, for Mn-Co-Ge-In/Al_2_O_3_ and Mn-Co-Ge-In/SrTiO_3_, the NTE coefficients are −6.56 × 10^−6^/K (from 270 K to 390 K) and −4.82 × 10^−6^/K (from 290 K to 390 K), respectively. These values are a bit smaller in comparison to those of the well-known commercial NTE materials ZrW_2_O_8_ (−9 × 10^−6^/K). Otherwise, the Mn-Co-Ge-In/LAO with the smallest island size denotes near-zero CTE of −2 × 10^−6^/K in the temperature range from 290 to 390 K.

The deposition of films completely overcomes the brittleness nature of Mn-Co-Ge-In alloy and much more durable. Repeated experiments demonstrated that the NTE behavior is completely repeatable between different cycles for the all films (Figure 12). All the results denotes that durable thin films with different CTE can be designed through choosing appropriate substrates or introducing a buffer layer, which would be quite useful for practical applications.

#### Negative thermal expansion in Fe-doped MnNiGe composites

Similar to MnCoGe-based alloys, MnNiGe-based alloys also denote NTE due to the martensitic transition from Ni_2_In-type hexagonal to TiNiSi-type orthorhombic structure. Still, Fe-doped MnNiGe alloys naturally crack into small pieces after annealing. Recently, by preparing the alloy through solid state interaction, Zhao et al. (2018) reported the NTE of MnNiGe-based alloy. The average CTE of Mn_0.9_Fe_0.1_NiGe alloy with Fe replacing Mn reaches a giant value of α_L_ ~ −285.23 × 10^−6^ K in a temperature window from 192 to 305 K (ΔT = 113 K), while the α_L_ of MnNi_0.9_Fe_0.1_Ge alloy with Fe replacing Ni reaches −1,167 × 10^−6^/K in a narrow temperature window (246–305 K, ΔT = 59 K). The introduction of Fe changes the magnetic state of martensitic phase from the spiral AFM structure into FM state.

**Figure 13:**
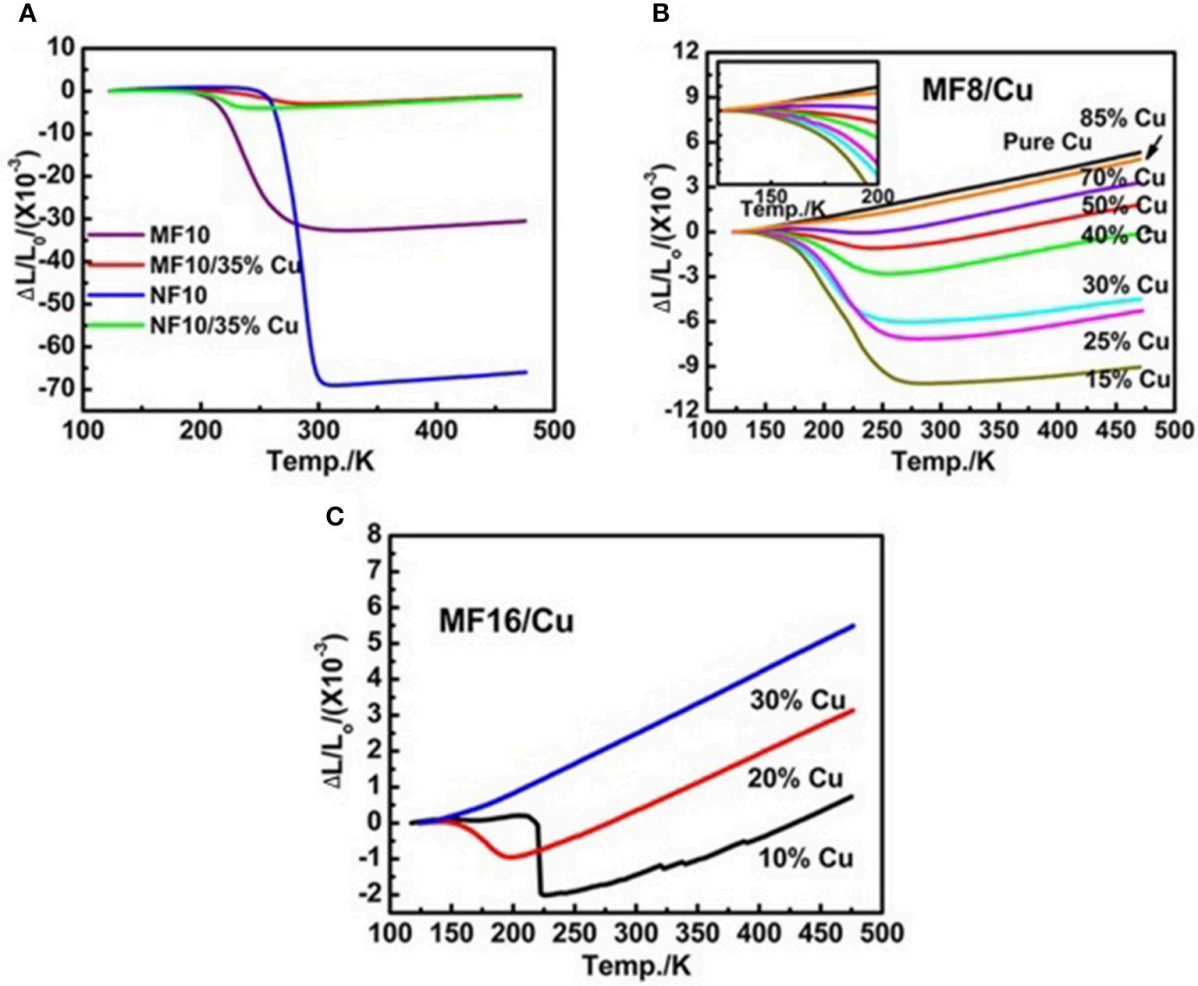
The temperature dependence of ΔL/L_0_ curves of **(A)** Mn_0.9_Fe_0.1_NiGe, MnNi_0.9_Fe_0.1_Ge, Mn_0.9_Fe_0.1_NiGe/35%Cu and MnNi_0.9_Fe_0.1_Ge/35%Cu, **(B)** MnNi_0.92_Fe_0.08_Ge/*x*%Cu (*x* = 15, 25, 30, 40, 50, 70, 85, 100), and **(C)** MnNi_0.94_Fe_0.16_Ge /*x*%Cu (*x* = 10, 20, 30), where the L_0_ is the length of the sample at 123 K. Reproduced under a Creative Commons Attribution 4.0 International License from (Zhao et al., 2018).

Furthermore, commercial Cu powders with positive thermal expansion has been chosen as the matrix metal, both acting as the compensators to control CTE and bonder to increase the mechanical properties of the alloy. The study (Zhao et al., 2018) revealed that the introduced Cu powers rarely react with the Fe-doped MnNiGe alloy. The cracks and holes can be filled by Cu particles, and the resulted MnNiGe/*x*%Cu composites is more compact with increasing Cu contents. The CTE of both Mn_0.9_Fe_0.1_NiGe and MnNi_0.9_Fe_0.1_Ge alloy decreases by introducing 35% Cu, as shown in Figure 13A. The linear thermal expansion behavior of Mn_0.92_Fe_0.08_NiGe/*x*% Cu and Mn_0.84_Fe_0.16_NiGe/*x*% Cu are presented in Figures 13B,C. The absolute CTE values of the two serials of composites decrease and the NTE temperature windows become narrow with increasing the Cu ratio. Increasing the Cu content to 70%, the composites display near-ZTE behaviors due to the compensation effect between the PTE properties of Cu matrix and the NTE properties of Fe-doped MnNiGe alloys. A low thermal expansion behavior with a CTE α_L_ ~ −4.73 × 10^−6^/K was observed in a temperature range from 173 to 229 K (ΔT = 56 K). In MnNi_0.92_Fe_0.08_/10%Cu composite, a low α_L_ ~1.16 × 10^−6^ /K is also achieved in a wide temperature range from 125 to 215 K (ΔT = 90 K).

In addition, the mechanical performance can be enhanced for the Fe-doped MnNiGe compounds with Cu as binders. All these suggest the high potential of the composites as NTE materials.

## RC_o2_ compounds

### Crystal structure

**Figure 14:**
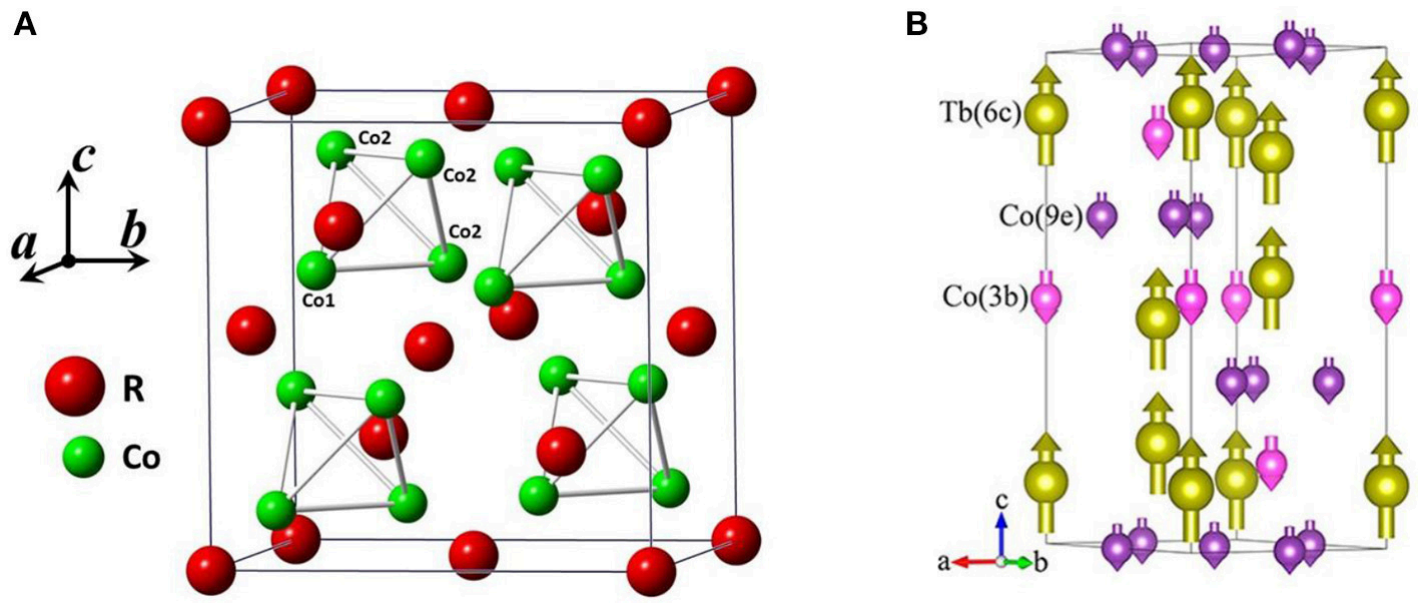
**(A)** Schematic of crystal structure for RCo_2_ cubic Laves phase(space group Fd$\bar{3}$m). **(B)** Crystal and magnetic structure of TbCo_1.9_Fe_0.1_ compound with rhombohedral distortion (space group R$\bar{3}$m) at 10 K. **(B)** reprinted with permission from (Song et al., 2018). Copyright 2018, ACS Publishing Limited.

The cubic Laves phases RCo_2_ (R = rare earth elements) have intensively been studied for the rich magnetic properties, which arise from the highly localized 4f electrons of rare earth elements and the interactions with the itinerant 3d electrons of the transition elements. For the RCo_2_ with R = Nd, Pr, Sm, Gd, Tb, Tm, the magnetic transition is of second-order in nature, while for R = Dy, Ho, and Er, the magnetic transition becomes of first-order (Khmelevskyi and Mohn, 2000). RCo_2_ compounds have a cubic Laves C15 structure with space group Fd$\bar{3}$m around room temperature. In the cubic C15 structure, rare atoms locate at sites 8(a) (0.125, 0.125, 0.125) and Co atoms at sites 16(d) (0.5, 0.5, 0.5) (Figure 14A; Ouyang et al., 2005a). With temperature decreasing, ErCo_2_ and TbCo_2_ exhibit a rhombohedral distortion (space group R$\bar{3}$m) below Curie temperature T_C_ (Ouyang et al., 2005a; Kozlenko et al., 2015). At low temperature, Er (Tb), Co1, and Co2 atoms occupy Wyckoff sites 6c (0, 0, z), 3b (0, 0, 0.5) and 9e (0.5, 0, 0), respectively. Figure 14B shows the crystal and magnetic structure of the TbCo_1.9_Fe_0.1_ compound at 10 K, the moments of Tb and Co atoms are antiparallel alignment along the c axis, which is similar to that of ErCo_2_ and TbCo_2_. However, NdCo_2_ and HoCo_2_ (Ouyang et al., 2005b; Mudryk et al., 2016) undergo two crystallographic transitions: the room temperature cubic lattice (space group Fd$\bar{3}$m) transforms into the tetragonal one (I4_1_/amd) at T_C_, and then becomes orthorhombic (space group: Fddd) below spin orientation T_SR_.

### Magnetocaloric effect

Numerous studies have been carried out on phase transition and magnetocaloric effect (MCE) for RCo_2_ compounds (Wada et al., 1999, 2001; Duc et al., 2002; Gomes et al., 2002; Wang et al., 2002a,b, 2003; Liu and Altounian, 2005). Experimental and theoretical investigations show that the compounds with R = Er, Ho, and Dy show giant MCE owing to the first-order nature of magnetic transition. In 2001, Wada et al. reported the MCE of single crystal ErCo_2_. The maximum magnetic entropy change, –ΔS, is as high as 10.6 J mol^−1^ K^−1^ (37.2 J kg^−1^ K^−1^) and the maximal adiabatic temperature change, ΔT_ad_, reaches ~9.2 K under a magnetic field change of 0–5 T (Wada et al., 1999). Owing to the large MCE, ErCo_2_ is usually considered to be a standard material for comparison in the low temperature region around 30 K. Furthermore, the maximum entropy change of DyCo_2_ and HoCo_2_ is 11 and 20 J kg^−1^ K^−1^ (Duc et al., 2002) under 0–5 T, respectively. The large MCE of RCo_2_ compounds is relevant to the large magnetic moment of R atoms, and more importantly, the crystallographic transition with negative volume expansion of different amplitudes also plays a key role for the various MCE. A small magnetic moment is induced in Co atoms, which is usually antiparallel to R atoms, resulting in the ferrimagnetic nature of the compounds.

In addition, some studies indicated that the replacement of R atoms by other Lanthanide elements can regulate the phase transition and MCE. Liu et al. (Liu and Altounian, 2005) reported that the Curie temperature of (Er_1−x_Gd_x_)Co_2_ increases from 32 to 403 K with the substitution of Er by Gd. However, the maximum value of -ΔS under 0–5 T decreases from 15.3 J kg^−1^ K^−1^ at *x* = 0.1 to 3.2 J kg^−1^ K^−1^ at *x* = 0.6. Furthermore, it has been found that the substitution of Dy by Gd will rapidly reduce the MCE in DyCo_2_ (Wang et al., 2002b), but replacing Tb with Er increases the MCE of TbCo_2_ (Gomes et al., 2002). On the other hand, Wang et al. (2002a) reported that using 7% of Si to replace Co can increase the Curie temperature of DyCo_2_ up to 168 K, but weakens the first-order phase transition properties and causes a significant reduction in the magnetocaloric effect. It is worth mentioning that the replacement of Co by Ni lowers T_C_ of Ho(Co_1−x_Ni_x_)_2_, while the large MCE is retained up to *x* = 0.1 though the phase transition changed from first-order to the second-order in nature (Wada et al., 2001).

### Negative thermal expansion behaviors

In RCo_2_ compounds with R = Dy, Ho, and Er, the magnetic ordering around T_C_ is accompanied by negative thermal expansion of lattice. Recently, Song et al. reported a wide temperature ZTE in the Tb(Co, Fe)_2_ and found a negligible thermal expansion coefficient, α_L_, in TbCo_1.9_Fe_0.1_, which is nearly two orders smaller than that of the common alloys (Song et al., 2018).

**Figure 15:**
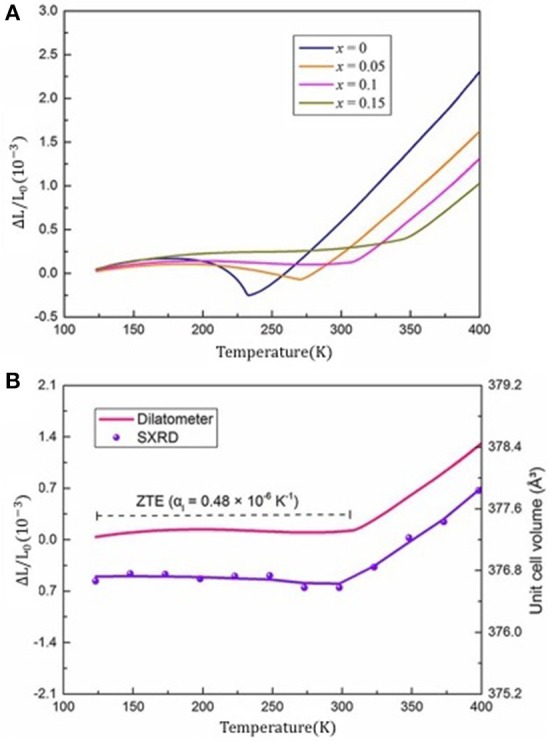
**(A)** Temperature dependent linear thermal expansion (ΔL/L_0_) of Tb(Co_2−x_Fe_x_). **(B)** The ZTE in the TbCo_1.9_Fe_0.1_ measured by both dilatometer and SXRD. Reprinted with permission from (Song et al., 2018). Copyright 2018, ACS Publishing Limited.

Figure 15A displays the temperature dependent linear thermal expansion (ΔL/L_0_) for Tb(Co_2−x_Fe_x_) compounds. It is found that the NTE and PTE can be obtained by adjusting the Fe concentration in Tb(Co_2−x_Fe_x_). Remarkably, the TbCo_1.9_Fe_0.1_ compound exhibits a ZTE property over a wide temperature range from 123 to 307 K. The average linear CTE of Tb(Co_1.9_Fe_0.1_) is α_L_ = 0.48 × 10^−6^ K^−1^ and intrinsic unit cell also confirmed the reliability of ZTE which was determined by SXRD (synchrotron X-ray diffraction), as shown in Figure 15B. This CTE of Tb(Co_1.9_Fe_0.1_) is about two orders of magnitude smaller than that of the Al (α_L_ = 23.1 × 10^−6^ K^−1^) and Cu (α_L_ = 17.7 × 10^−6^K^−1^). Furthermore, this value is also smaller than the CTE of Invar alloys with composition Fe_0.65_Ni_0.35_ (α_L_ = 1.5 × 10^−6^ K^−1^, 193–373 K) (Guillaume, 1897).

The magnetic moment of Tb atoms, M_Tb_, plays a dominationg role in the ferrimagnetic nature of the Tb(Co_2−x_Fe_x_) compounds. The spontaneous volume magnetostriction (ω_s_) was used to quantitatively describe the contribution of magentovolume effect to the abnormal change of lattice. For TbCo_1.9_Fe_0.1_, and the ω_s_ is denoted by ω_s_ = ω_exp_-ω_nm_,where ω_exp_ is the unit cell volume obtained by NPD, and ω_nm_ is calculated based on the Debye-Grüneisen relationship. A strong correlation between M${}_{\text{Tb}}^{2}$ and ω_s_ was denoted by the equation ω_s_(T) = k C M(T)^2^, where k and C represent compressibility and magnetovolume coupling constant, and M(T) the amplitudes of magnetization (Gruneisen and Goens, 1926; Sayetat et al., 1998). That is to say, the spontaneous magnetostriction is closely related to the magnetization. The decrease of magnetization with temperature is responsible for the negative contribution to volume expansion, which neutralizes the normal lattice expansion, and hence produces the ZTE.

### A brief introduction on antiperovskite compounds

Antiperovskite compounds A_3_BC (A = Fe, Mn; B = Ga, Sn, Cu, Zr, Fe, Co; C = C, N, H) are well-known (Takenaka and Takagi, 2005; Iikubo et al., 2008; Sun et al., 2010a,b, 2012; Song et al., 2011; Wang et al., 2012; Lin et al., 2015; Shi et al., 2016, 2018; Guo et al., 2018) for the rich characteristics of NTE and ZTE, as well as the baromagnetic, and barocaloric properties, though the comprehensive performance of MCE is not as good as the conventional material Gd around room temperature. This material has a very large family, which shows rich magnetic and lattice structure depending on compositions and atomic occupations. Experts, such as C. Wang's group and Y.P. Sun's group, have done a lot of excellent studies in the antiperovskite compounds, particularly on the NTE properties. Moreover, fascinating properties were also reported in some compositions, such as baromagnetic (Shi et al., 2016), barocaloric effect caused by spin frustration (Matsunami et al., 2015), and large magnetocaloric effect (Sun et al., 2012). Specifically, C. Wang' group have reported a large amount of research work on NTE and MCE in antiperovskite compounds and also thought that lattice entropy is important to be excited due to large negative thermal expansion behavior driven by magnetic effects in the antiperovskite compounds so as to improve the magnetocaloric effect. A specific review article focusing on the antiperovskite compounds should be summarized separately, and we do not address more here.

## Conclusion

In summary, negative thermal expansion (NTE) behaviors in the materials with giant magnetocaloric effects (MCE) have been reviewed. Representative materials including La(Fe,Si)_13_, MM'X, and RCo_2_ have been included. Antiperovskite compounds are also briefly introduced. MCE materials are used for magnetic refrigeration while NTE materials for thermal compensation. Although the materials have distinct applications, the physical sources are similar particularly for the novel MCE and phase-transition-type NTE materials, both originating from the strong spin-lattice coupling. A number of MCE materials show abnormal lattice expansion owing to magnetic interactions, which provides a natural platform for exploring negative thermal expansion materials. The magnetostructural transformation in the giant MCE materials can be adjusted by utilizing its sensitivity to chemical/physical pressure, hence abnormal lattice expansion in an extended temperature range can be achieved. The present review provides implications for developing novel NTE materials and multifunctional devices.

## Author contributions

FH wrote the article in cooperation with FS, JH, and YL. All authors reviewed the manuscript.

### Conflict of interest statement

The authors declare that the research was conducted in the absence of any commercial or financial relationships that could be construed as a potential conflict of interest.

## References

[B1] Akkera H. S. Singh I. Kaur D. (2015). Martensitic phase transformation of magnetron sputtered nanostructured Ni–Mn–In ferromagnetic shape memory alloy thin films. J. Alloys Compd. 642, 53–62. 10.1016/j.jallcom.2015.03.261

[B2] Annaorazov M. P. Nikitin S. A. Tyurin A. L. Asatryan K. A. Dovletov A. (1996). Anomalously high entropy change in FeRh alloy. J. Appl. Phys. 79, 1689–1695. 10.1063/1.360955

[B3] Azuma M. Chen W. T. Seki H. Czapski M. Olga S. Oka K.. (2011). Colossal negative thermal expansion in BiNiO_3_ induced by intermetallic charge transfer. Nat. Commun. 2:347. 10.1038/ncomms1361 21673668 PMC3156814

[B4] Caron L. Trung N. T. Bruck E. (2011). Pressure-tuned magnetocaloric effect in Mn_0.93_Cr_0.07_CoGe. Phys. Rev. B 84, 020414– 020417. 10.1103/PhysRevB.84.020414

[B5] Chen J. Nittala K. Forrester J. S. Jones J. L. Deng J. X. Yu R. B.. (2011). The Role of Spontaneous Polarization in the Negative Thermal Expansion of Tetragonal PbTiO_3_-Based Compounds. J. Am. Chem. Soc. 133, 11114–11117. 10.1021/ja2046292 21696173

[B6] Chu X. X. Wu Z. X. Huang C. J. Huang R. J. Zhou Y. Li L. F. (2012). ZrW_2_O_8_-doped epoxy as low thermal expansion insulating materials for superconducting feeder system. Cryogenics 52, 638–641. 10.1016/j.cryogenics.2012.04.016

[B7] Du Z. C. Zhu M. R. Wang Z. G. Yang J. G. (2016). Design and application of composite platform with extreme low thermal deformation for satellite. Compos. Struct. 152, 693–703. 10.1016/j.compstruct.2016.05.073

[B8] Duc N. H. Anh D. T. K. Brommer P. E. (2002). Metamagnetism, giant magnetoresistance and magnetocaloric effects in RCo_2_-based compounds in the vicinity of the Curie temperature. Phys. B 319, 1–8. 10.1016/S0921-4526(02)01099-2

[B9] Fujita A. Fujieda S. Hasegawa S. Fukamichi K. (2003). Itinerant-electron metamagnetic transition and large magnetocaloric effects in La(Fe_x_Si_1−x_)_13_ compounds and their hydrides. Phys. Rev. B 67:104416 10.1103/PhysRevB.67.104416

[B10] Gercsi Z. Hono K. Sandeman K. G. (2011). Designed metamagnetism in CoMnGe_1−x_P_x_. Phys. Rev. B 83:174403 10.1103/PhysRevB.83.174403

[B11] Gomes A. M. Reis M. S. Oliveira I. S. Guimar A. P. Takeuchi A. Y. (2002). Magnetocaloric effect in (Er, Tb)Co_2_. J. Magn. Magn. Mater. 242 870–872. 10.1016/S0304-8853(01)01327-0

[B12] Gruneisen E. Goens E. (1926). Tests on metal crystals IV thermo electrical characteristics of zinc and cadmium. Z. Phys. 37, 278–291. 10.1007/BF01397101

[B13] Gschneidner K. A. Pecharsky A. Dennis K. W. (1997). Some observations on the Gd-rich side of the Gd-C system. J. Alloys Compd. 260, 107–110. 10.1016/S0925-8388(97)00146-1

[B14] Gschneidner K. A. Jr. Pecharsky V. K. Tsokol A. O. (2005). Recent developments in magnetocaloric materials. Rep. Prog. Phys. 68, 1479–1539. 10.1088/0034-4885/68/6/R04

[B15] Guillaume C. É. (1897). Investigations on Nickel and its alloys. CR. Acad. Sci. 125:18.

[B16] Guo X. G. Tong P. Lin J. C. Yang C. Zhang K. Lin S.. (2018). Effects of Cr substitution on negative thermal expansion and magnetic properties of antiperovskite Ga1–xCrxN0.83Mn3 compounds. Front. Chem. 6:75. 10.3389/fchem.2018.00075 29619367 PMC5871658

[B17] Hu F. X. (2002). Magnetic Properties and Magnetic Entropy Change of Fe-Based La(Fe,M)13 Compounds and Ni-Mn-Ga Alloys. Ph. D thesis, Institute of Physics, Chinese academy of Sciences.

[B18] Hu F. X. Gao J. Qian X. L. Ilyn M. Tishin A. M. Sun J. R. (2005). Magnetocaloric effect in itinerant electron metamagnetic systems La(Fe_1−x_Cox)_11.9_Si_1.1_. J. Appl. Phys. 97:10M303. 10.1063/1.1847071

[B19] Hu F. X. Shen B. G. Sun J. R. (2000a). Magnetic entropy change in Ni_51.5_Mn_22.7_Ga_25.8_ alloy. Appl. Phys. Lett. 76, 3460–3462. 10.1063/1.126677

[B20] Hu F. X. Shen B. G. Sun J. R. Cheng Z. H. Rao G. H. Zhang X. X. (2001). Influence of negative lattice expansion and metamagnetic transition on magnetic entropy change in the compound LaFe_11.4_Si_1.6_. Appl. Phys. Lett. 78, 3675–3677. 10.1063/1.1375836

[B21] Hu F. X. Shen B. G. Sun J. R. Wang G. J. Cheng Z. H. (2002). Very large magnetic entropy change near room temperature in LaFe_11.2_Co_0.7_Si_1.1_. Appl. Phy. Lett. 80, 826–828. 10.1063/1.1447592

[B22] Hu F. X. Shen B. G. Sun J. R. Zhang X. X. (2000b). Great magnetic entropy change in La (Fe, M)_13_ (M = Si, Al) with Co doping. Chin. Phys. 9, 550–553. 10.1088/1009-1963/9/7/016

[B23] Huang R. J. Liu Y. Y. Fan W. Tan J. Xiao F. R. Qian L. H.. (2013). Giant negative thermal expansion in NaZn_13_-Type La(Fe, Si, Co)_13_ compounds. J. Am. Chem. Soc. 135, 11469–11472. 10.1021/ja405161z 23885928

[B24] Iikubo S. Kodama K. Takenaka K. Takagi H. Takigawa M. Shamoto S. (2008). Local lattice distortion in the giant negative thermal expansion material Mn_3_Cu_1−x_Ge_x_N. Phys. Rev. Lett. 101:205901. 10.1103/PhysRevLett.101.205901 19113356

[B25] Khmelevskyi S. Mohn P. (2000). Order of the magnetic phase transitions in RCo_2_ (R = rare earth) intermetallic compounds. J. Phys. Condens. Matter. 12, 9453–9464. 10.1088/0953-8984/12/45/308

[B26] Kozlenko D. P. Burzo E. Vlaic P. Kichanov S. E. Rutkauskas A. V. Savenko B. N. (2015). Sequential cobalt magnetization collapse in ErCo_2_: beyond the limits of itinerant electron metamagnetism. Sci. Rep. 5:8620. 10.1038/srep08620 25727134 PMC4345330

[B27] Krenke T. Duman E. Acet M. Wassermann E. F. Moya X. Manosa L.. (2005). Inverse magnetocaloric effect in ferromagnetic Ni-Mn-Sn alloys. Nature Mater. 4, 450–454. 10.1038/nmat1395 15895096

[B28] Krypiakewytsch P. I. Zaretsch O. S. Hladysch E. I. Bodak O. I. (1968). Ternary compounds of NaZn_13_ type. Z. Anorg. Allg. Chem. 358:90 10.1002/zaac.19683580110

[B29] Li S. P. Huang R. J. Zhao Y. Q. Wang W. Han Y. M. Li L. F. (2017). Zero thermal expansion achieved by an electrolytic hydriding method in La(Fe, Si)_13_ compounds. Adv. Func. Mater. 27:1604195 10.1002/adfm.201604195

[B30] Lin J. C. Tong P. Tong W. Lin S. Wang B. S. Song W. H. (2015). Tunable negative thermal expansion related with the gradual evolution of antiferromagnetic ordering in antiperovskite manganese nitrides Ag1-xNMn3+x (0 ≤ x ≤ 0.6). Appl. Phys. Lett. 106:082405 10.1063/1.4913663

[B31] Lin J. C. Tong P. Zhang K. Tong H. Y. Guo X. G. Yang C. (2016). Colossal negative thermal expansion with an extended temperature interval covering room temperature in fine-powdered Mn_0.98_CoGe. Appl. Phy. Lett. 109:241903 10.1063/1.4972234

[B32] Liu D. M. Huang Q. Z. Yue M. Lynn J. W. Liu L. J. Chen Y. (2009). Temperature, magnetic field, and pressure dependence of the crystal and magnetic structures of the magnetocaloric compound Mn1.1Fe0.9(P0.8Ge0.2). Phys. Rev. B 80, 174415 10.1103/PhysRevB.80.174415

[B33] Liu E. K. Wang W. H. Feng L. Zhu W. Li G. J. Chen J. L.. (2012). Stable magnetostructural coupling with tunable magnetoresponsive effects in hexagonal ferromagnets. Nat. Commun. 3:873. 10.1038/ncomms1868 22643900

[B34] Liu E. K. Zhu W. Feng L. Chen J. L. Wang W. H. Wu G. H. (2010). Vacancy-tuned paramagnetic/ferromagnetic martensitic transformation in Mn-poor Mn_1−x_CoGe alloys, Europhys. Lett. 91:17003 10.1209/0295-5075/91/17003

[B35] Liu J. Gottschall T. Skokov K. P. Moore J. D. Gutfleisch O. (2012). Giant magnetocaloric effect driven by structural transitions. Nat. Mater. 11, 620–626. 10.1038/NMAT3334 22635044

[B36] Liu X. B. Altounian Z. (2005). Magnetocaloric effect in (Er_1−x_Gd_x_)Co_2_ pseudobinary compounds. J. Magn. Magn. Mater. 292, 83–88. 10.1016/j.jmmm.2004.10.100

[B37] Liu Y. Qiao K. M. Zuo S. L. Zhang H. R. Kuang H. Wang J. (2018). Negative thermal expansion and magnetocaloric effect in Mn-Co-Ge-In thin films. Appl. Phys. Lett. 112:012401 10.1063/1.5009985

[B38] Liu Y. Shen F. R. Zhang M. Bao L. F. Wu R. R. Zhao Y. Y. (2015). Stress modulated martensitic transition and magnetocaloric effect in hexagonal Ni_2_In-type MnCoGe_1−−x_In_x_ alloys. J. Alloys Compd. 649, 1048–1052. 10.1016/j.jallcom.2015.07.234

[B39] Mary T. A. Evans J. S. O. Vogt T. Sleight A. W. (1996). Negative thermal expansion from 0.3 to 1050 Kelvin in ZrW_2_O_8_. Science 272, 90–92. 10.1126/science.272.5258.90

[B40] Matsunami D. Fujita A. Takenaka K. Kano M. (2015). Giant barocaloric effect enhanced by the frustration of the antiferromagnetic phase in Mn3GaN. Nature Mater. 14, 73–78. 10.1038/NMAT4117 25344781

[B41] Morellon L. Blasco J. Algarabel P. A. Ibarra M. R. (2000). Nature of the first-order antiferromagnetic-ferromagnetic transition in the Ge-rich magnetocaloric compounds Gd-5(SixGe1-x)(4). Phys. Rev. B 62, 1022–1026. 10.1103/PhysRevB.62.1022

[B42] Mosca D. H. Vidal F. Etgens V. H. (2008). Strain engineering of the magnetocaloric effect in MnAs epilayers. Phys. Rev. Lett. 101:125503. 10.1103/PhysRevLett.101.125503 18851386

[B43] Mudryk Y. Paudyal D. Pathak A. K. Pecharsky V. K. Gschneidner K. A. (2016). Balancing structural distortions via competing 4f and itinerant interactions: a case of polymorphism in magnetocaloric HoCo_2_. J. Mater. Chem. C 4, 4521–4531. 10.1039/c6tc00867d

[B44] Namba Y. Takehara H. Nagano Y. (2001). Fracture strength of zero-thermal-expansion glass-ceramics for ultra-precision components. CIRP Ann. Manuf. Technol. 50, 239–242. 10.1016/S0007-8506(07)62113-1

[B45] Niziol S. Weselucha A. Bazela W. Szytula A. (1981). Magnetic properties of the Co_x_Ni_1−−x_MnGe system. Solid State Commun. 39, 1081–1085. 10.1016/0038-1098(81)90213-1

[B46] Ouyang Z. W. Wang F. W. Hang Q. Liu W. F. Liu G. Y. Lynn J. W. (2005a). Temperature dependent neutron powder diffraction study of the Laves phase compound TbCo_2_. J. Alloys Compd. 390, 21–25. 10.1016/j.jallcom.2004.08.028

[B47] Ouyang Z. W. Wang F. W. Huang Q. Liu W. F. Xiao Y. G. Lynn J. W. (2005b). Magnetic structure, magnetostriction, and magnetic transitions of the Laves-phase compound NdCo_2_. Phys. Rev. B 71:064405 10.1103/PhysRevB.71.064405

[B48] Pecharsky V. K. Gschneidner K. A. Jr. (1997). The giant magnetocaloric effect in Gd_5_(Si_2_Ge_2_). Phys. Rev. Lett. 78, 4494–4497. 10.1103/PhysRevLett.78.4494 10990754

[B49] Pires A. L. Belo J. H. Gomes L. Hadimani R. L. Jiles D. C. Fernandes L. (2015). Annealing influence on the magnetostructural transition in Gd_5_Si_1.3_Ge_2.7_ thin films. Mater. Lett. 159, 301–304. 10.1016/j.matlet.2015.05.029

[B50] Rathmann C. L. Mann G. H. Nordberg M. E. (1968). A new ultralow-expansion, modified fused-silica glass. Appl. Opt. 7:819. 10.1364/AO.7.000819 20068691

[B51] Sayetat F. Fertey P. Kessler M. (1998). An easy method for the determination of debye temperature from thermal expansion analyses. J. Appl. Crystallogr. 31, 121–127. 10.1107/S0021889897006936

[B52] Shen B. G. Sun J. R. Hu F. X. Zhang H. W. Cheng Z. H. (2009). Recent progress in exploring magnetocaloric materials. Adv. Mater. 21, 4545–4564. 10.1002/adma.200901072

[B53] Shen F. R. Kuang H. Hu F. X. Wu H. Huang Q. Z. Liang F. X. (2017). Ultra-low thermal expansion realized in giant negative thermal expansion materials through self-compensation. APL Mater. 5:106102 10.1063/1.4990481

[B54] Shi K. W. Sun Y. Colin C. V. Wang L. Yan J. Deng S. H. (2018). Investigation of the spin-lattice coupling in Mn3Ga1-xSnxN antiperovskites. Phys. Rev. B 97:054110 10.1103/PhysRevB.97.054110

[B55] Shi K. W. Sun Y. Yan J. Deng S. H. Wang L. Wu H.. (2016). Baromagnetic effect in antiperovskite Mn3Ga0.95N0.94 by neutron powder diffraction analysis. Adv. Mater. 28, 3761–3767. 10.1002/adma.201600310 27007214

[B56] Song X. Y. Sun Z. H. Huang Q. Z. Rettenmayr M. Liu X. M. Seyring M.. (2011). Adjustable zero thermal expansion in antiperovskite manganese nitride. Adv. Mater. 23:4690. 10.1002/adma.201102552 21913237

[B57] Song Y. Z. Chen J. Liu X. Z. Wang C. W. Zhang J. Liu H.. (2018). Zero thermal expansion in magnetic and metallic Tb(Co, Fe)_2_ intermetallic compounds. J. Am. Chem. Soc. 140, 602–605. 10.1021/jacs.7b12235 29292996

[B58] Sullivan L. M. Lukehart C. M. (2005). Zirconium tungstate (ZrW_2_O_8_)/polyimide nanocomposites exhibiting reduced coefficient of thermal expansion. Chem. Mater. 17, 2136–2141. 10.1021/cm0482737

[B59] Sun Y. Wang C. Huang Q. Z. Guo Y. F. Chu L. H. Arai M.. (2012). Neutron diffraction study of unusual phase separation in the antiperovskite nitride Mn3ZnN. Inorg. Chem. 51, 7232–7236. 10.1021/ic300978x 22720658

[B60] Sun Y. Wang C. Wen Y. C. Chu L. H. Man N. (2010a). Negative thermal expansion and correlated magnetic and electrical properties of Si-Doped Mn3GaN compounds. J. Am. Ceram. Soc. 93, 650–653. 10.1111/j.1551-2916.2009.03482.x

[B61] Sun Y. Wang C. Wen Y. C. Chu L. H. Pan H. Niez M. (2010b). Negative thermal expansion and magnetic transition in anti-perovskite structured Mn3Zn1-xSnxN compounds. J. Am. Ceram. Soc. 93, 2178–2181. 10.1111/j.1551-2916.2010.03711.x

[B62] Takenaka K. Asano K. Misawa M. Takagi H. (2008). Negative thermal expansion in Ge-free antiperovskite manganese nitrides: Tin-doping effect. Appl. Phys. Lett. 92:011927 10.1063/1.2831715

[B63] Takenaka K. Hamada T. Kasugai D. Sugimoto N. (2012). Tailoring thermal expansion in metal matrix composites blended by antiperovskite manganese nitrides exhibiting giant negative thermal expansion. J. Appl. Phys. 112:083517 10.1063/1.4759121

[B64] Takenaka K. Ichigo M. (2014). Thermal expansion adjustable polymer matrix composites with giant negative thermal expansion filler. Compos. Sci. Technol. 104, 47–51. 10.1016/j.compscitech.2014.08.029

[B65] Takenaka K. Okamoto Y. Shinoda T. Katayama N. Sakai Y. (2017). Colossal negative thermal expansion in reduced layered ruthenate. Nat. Commun. 8:14102. 10.1038/ncomms14102 28071647 PMC5234094

[B66] Takenaka K. Takagi H. (2005). Giant negative thermal expansion in Ge-doped anti-perovskite manganese nitrides. Appl. Phys. Lett. 87:261902 10.1063/1.2147726

[B67] Tegus O. Brück E. Buschow K. H. J. de Boer F. R. (2002). Transition-metal-based magnetic refrigerants for room-temperature applications. Nature 415, 150–152. 10.1038/415150a 11805828

[B68] Wada H. Tanabe Y. (2001). Giant magnetocaloric effect of MnAs_1−x_Sb_x_. Appl. Phys. Lett. 79, 3302–3304. 10.1063/1.1419048

[B69] Wada H. Tanabe Y. Shiga M. Sugawara H. Sato H. (2001). Magnetocaloric effects of Laves phase Er(Co_1−x_Ni_x_)_2_ compounds. J. Alloys Compd. 316, 245–249. 10.1016/S0925-8388(00)01305-0

[B70] Wada H. Tomekawa S. Shiga M. (1999). Magnetocaloric properties of a first-order magnetic transition system ErCo_2_. Cryogenics 39, 915–919. 10.1016/S0011-2275(99)00121-6

[B71] Wang C. Chu L. H. Yao Q. R. Sun Y. Wu M. M. Ding L. (2012). Tuning the range, magnitude, and sign of the thermal expansion in intermetallic Mn3(Zn, M)x N(M = Ag, Ge). Phys. Rev. B 85:220103 10.1103/PhysRevB.85.220103

[B72] Wang D. H. Liu H. D. Tang S. L. Tang T. Wen J. F. Du Y. W. (2002a). Low-field magnetic entropy change in Dy(Co_1−x_Si_x_)_2_. Solid State Commun. 121, 199–202. 10.1016/S0038-1098(01)00486-0

[B73] Wang D. H. Liu H. D. Tang S. L. Yang S. Huang S. L. Du Y. W. (2002b). Magnetic properties and magnetocaloric effects in (Gd_x_Dy_1−x_)Co_2_ compounds. Phys. Lett. A 297, 247–252. 10.1016/S0375-9601(02)00159-7

[B74] Wang D. H. Tang S. L. Huang S. L. Su Z. H. Han Z. D. Du Y. W. (2003). The origin of the large magnetocaloric effect in RCo_2_ (R = Er, Ho and Dy). J. Alloy. Compd. 360, 11–13. 10.1016/S0925-8388(03)00324-4

[B75] Wang J. T. Wang D. S. Chen C. F. Nashima O. Kanomata T. Mizuseki H. (2006). Vacancy induced structural and magnetic transition in MnCo_1−x_Ge. Appl. phys. Lett. 89, 262504–262506. 10.1063/1.2424273

[B76] Wu R. R. Bao L. F. Hu F. X. Wu H. Huang Q. Z. Wang J.. (2015). Giant barocaloric effect in hexagonal Ni_2_In-type Mn-Co-Ge-In compounds around room temperature. Sci. Rep. 5:18027. 10.1038/srep18027 26673677 PMC4682185

[B77] Wu R. R. Shen F. R. Hu F. X. Wang J. Bao L. F. Zhang L. (2016). Critical dependence of magnetostructural coupling and magnetocaloric effect on particle size in Mn-Fe-Ni-Ge compounds. Sci. Rep. 6:20993 10.1038/srep20993 26883719 PMC4756685

[B78] Zhao W. J. Sun Y. Liu Y. F. Shi K. W. Lu H. Q. Song P.. (2018). Negative thermal expansion over a wide temperature range in Fe-doped MnNiGe composites. Front. Chem. 6:15. 10.3389/fchem.2018.00015 29468152 PMC5808177

[B79] Zhao Y. Y. Hu F. X. Bao L. F. Wang J. Wu H. Huang Q. Z.. (2015). Giant negative thermal expansion in bonded MnCoGe-based compounds with Ni_2_In-type hexagonal structure. J. Am. Chem. Soc. 137, 1746–1749. 10.1021/ja510693a 25629796

[B80] Zheng X. G. Kubozono H. Yamada H. Kato K. Ishiwata Y. Xu C. N. (2008). Giant negative thermal expansion in magnetic nanocrystals. Nat. Nanotechnol. 3, 724–726. 10.1038/nnano.2008.309 19057591

[B81] Zhou T. J. Cher M. K. Shen L. Hu J. F. Yuan Z. M. (2013). On the origin of giant magnetocaloric effect and thermal hysteresis in multifunctional alpha-FeRh thin films. Phys. Lett. A 377, 3052–3059. 10.1016/j.physleta.2013.09.027

